# Quality and reliability of cancer- and tumor-related videos on YouTube: a scoping review

**DOI:** 10.3389/fpubh.2026.1940232

**Published:** 2026-10-14

**Authors:** Jianjie Zhang, Lu Xu, Yu Fu, Yajuan Wang, Fangping Shi, Tao Shi

**Affiliations:** 1 School of Nursing, Kunming Medical University, Kunming, Yunnan, China; 2 The Third Affiliated Hospital of Kunming Medical University, Kunming, China; 3 Department of Thoracic Surgery and Urology, Luxi County People’s Hospital, Luxi, Yunnan, China; 4 Yunnan Cancer Hospital, Kunming, China; 5 Peking University Cancer Hospital Yunnan, Kunming, China

**Keywords:** cancer, health information, scoping review, video quality, YouTube

## Abstract

**Background:**

Cancer imposes a heavy burden on society, and YouTube has become an important channel for the public to access health information; however, the quality of cancer-related videos on YouTube varies widely. Furthermore, there is currently a lack of systematic reviews on the quality of cancer-related videos on this platform.

**Objectives:**

To systematically review the current state of research on the evaluation of the quality and reliability of cancer- and tumor-related videos on YouTube; to analyze trends in video quality, patterns of evaluation tool usage, the impact of uploader types on quality, and the relationship between quality and popularity.

**Methods:**

A scoping review approach was adopted. We conducted a systematic search of PubMed, EMBASE, Web of Science, the Cochrane Library, Scopus, CINAHL, CNKI, and the VIP database, covering the period from the inception of each database to June 1, 2026. We included original studies that evaluated the quality of cancer- and tumor-related videos on YouTube. Two researchers independently screened the literature and extracted data, with an additional independent reviewer assisting in the decision-making process.

**Results:**

A total of 54 studies were included, collectively analyzing a cumulative total of 5,051 individual video observations across 21 cancer-related topics. The proportion of studies reporting high-quality video samples increased descriptively from 0% in 2018–2019 to 31.3% in 2023–2025. GQS (35 instances), original DISCERN (27 instances), modified DISCERN (21 instances), and JAMA (18 instances) were the most commonly used evaluation tools. Most studies comparing uploader types reported higher quality scores for videos from healthcare professionals or institutions.

**Conclusion:**

The overall quality of cancer- and tumor-related videos on YouTube was generally low. The proportion of studies reporting moderate- or high-quality video samples was descriptively greater in more recent periods. Most studies comparing uploader types reported higher quality scores for videos from healthcare professionals or institutions. Greater professional involvement and platform-level strategies to identify and promote trustworthy health information may improve the educational value of these videos.

## 1 Introduction

Cancer has become one of the most serious public health issues worldwide, imposing a substantial disease burden. The annual number of new cancer cases worldwide is projected to reach approximately 34.4 million by 2050, representing a 67% increase from 2024 (1). Cancer patients have multi-level information needs during the diagnosis, treatment, and recovery phases. In the early stages following diagnosis, patients need to understand basic information about the disease, its staging, and treatment options to help alleviate the psychological impact (2); during treatment, they need to understand the benefits and risks of different treatment regimens as well as how to manage adverse reactions (3, 4); and during the rehabilitation phase, they need access to information on recurrence risk stratification, functional recovery, and psychosocial support to cope with the uncertainties associated with long-term survival (5, 6). Due to limitations in the time available for clinical communication and the continuity of follow-up care, these needs are difficult to fully meet, prompting patients and their families to turn to the internet for information (7–9). Social media has also become an important channel for patients, families, healthcare professionals, and other stakeholders to access and share information about cancer (10). Some studies even show that online platforms have become the primary source of health information for cancer patients (11).

Among various online media, videos—with their combination of audio and visuals, bite-sized content, and ease of understanding—have significantly lowered the barrier to accessing health information and have become an important channel for the public to obtain such information (10, 12). As the world’s second-most-visited website, with over 2 billion monthly active users, YouTube offers a wealth of video resources covering professional medical information and exerts significant influence on the global dissemination of health information (13). Compared to TikTok and Instagram, which primarily feature short videos, YouTube can accommodate videos of varying lengths, providing more ample space for the platform to present complex information on cancer screening, diagnosis, treatment, and recovery (14, 15). However, the low barrier to content publication and limited moderation also increase the risk of spreading inaccurate or misleading health information (16). Inaccurate or misleading information may interfere with patients’ treatment decisions, adversely affecting their treatment process and outcomes (17). Consequently, existing studies have conducted macro-level assessments of the quality of health information on YouTube (18), and some scholars have focused on video quality for specific diseases or populations [such as surgical patients (19)]. Related evaluations have also expanded into the field of rehabilitation, covering topics such as cardiac rehabilitation, exercise for ankylosing spondylitis, and stroke rehabilitation (20–23). However, previous reviews have typically covered a variety of new media formats rather than focusing specifically on YouTube videos about cancer, nor have they systematically synthesized evaluation tools, uploader types, temporal trends, and engagement metrics. Given the significant heterogeneity in relevant studies regarding cancer types, video sampling, evaluation tools, and outcome measures, a scoping review is appropriate for mapping the distribution of evidence, summarizing evaluation methods, and identifying research gaps. Therefore, this study systematically reviews the current state of research on the quality assessment of cancer-related videos on YouTube to provide a foundation for future research design and video quality evaluation.

## 2 Methods

This scoping review was conducted in accordance with the JBI methodology for scoping reviews and reported following the PRISMA Extension for Scoping Reviews (PRISMA-ScR).

### 2.1 Defining the research questions

This scoping review aims to systematically summarize the current state of research on the evaluation of the quality and reliability of cancer-related videos on the YouTube platform, focusing on answering the following questions: (1) What is the quality of these videos and how does it trend over time? (2) What is the relationship between uploader type and video quality? (3) What is the current status of research evaluation tools? (4) What are the patterns of correlation between video quality and dissemination metrics (views, likes, and comments)?

### 2.2 Inclusion and exclusion criteria for studies

Inclusion Criteria: ① The study platform is YouTube (other platforms may be included, provided that YouTube data can be extracted separately); ② Video content that addresses malignant tumors (cancer), benign tumors, borderline tumors, or tumors of undetermined nature; or clinical topics directly related to the prevention, screening, diagnosis, treatment, rehabilitation, and palliative care of the aforementioned conditions; ③ The study evaluates videos using a structured or validated quality assessment tool (e.g., DISCERN, GQS, PEMAT, JAMA, VIQI, etc.); ④ The study type is original research (cross-sectional studies, content analysis); ⑤ The full text is available through open access channels or institutional subscriptions;

Exclusion Criteria: ① Duplicate inclusions, or incomplete information and data;

### 2.3 Literature search

This study employed a search strategy combining subject headings with free-text terms to systematically search the following English-language databases: PubMed, EMBASE, Scopus, Web of Science, the Cochrane Library, and CINAHL. Chinese databases included China National Knowledge Infrastructure (CNKI) and VIP. The search used combinations of Chinese and English keywords such as “YouTube,” “cancer,” “tumor,” “video quality,” and “reliability assessment,” with a search time frame ranging from the inception of each database to June 1, 2026. No restrictions were imposed on the language of publication during the database search or literature screening processes (Supplementary Table 4).

### 2.4 Literature screening and data extraction

After importing the retrieved literature into EndNote software and removing duplicates, two researchers trained in evidence-based research methods independently screened the literature based on the inclusion and exclusion criteria. First, the titles and abstracts were reviewed; full texts of articles that appeared to meet the inclusion criteria were then read in full. At the same time, the reference lists of included studies were reviewed to identify and include any relevant studies that may have been overlooked. In the event of disagreement during the screening process, the matter was discussed and resolved with a third researcher to make a final decision on inclusion. Reasons for exclusion were documented for each excluded study.

Two researchers independently extracted data, which was then cross-checked upon completion. Microsoft Excel was used to summarize key information for each study: first author, video retrieval date, cancer type, video sample size, name of the evaluation tool, and main findings or conclusions (quality scores, comparisons of uploader types and quality, correlations between quality scores and dissemination metrics, self-reported study limitations, etc.). For studies that analyzed both YouTube and other platforms, only the independent data from the YouTube portion were extracted; if the data could not be separated, the study was excluded.

Because the evaluation tools and cutoff values used in the various studies differed, this review does not establish a uniform threshold or convert scores from different tools. The quality of video samples was primarily based on the original authors’ overall assessment, verified in conjunction with the scores and cutoff values they reported; when no overall assessment was provided, studies were classified according to the primary quality grade or the cutoff values used in the original study. Studies with inconsistent results or insufficient information were labeled as “mixed” or “unclassifiable” and were excluded from the calculation of quality proportions by time period.

To conduct a descriptive comparison of study results across different time periods, this review categorized studies based on the year of video retrieval into early (2018–2019), mid-period (2020–2022), and recent (2023–2025) periods. This grouping is used solely for narrative synthesis; no statistical methods were employed to determine the cutoff points. Studies that did not report the retrieval date were excluded from this comparison.

## 3 Results

### 3.1 Literature screening and basic characteristics

This study identified a total of 350 potentially relevant articles from English-language databases such as PubMed (*n* = 135), EMBASE (*n* = 34), Scopus (*n* = 73), Web of Science (*n* = 104), the Cochrane Library (*n* = 0), and CINAHL (*n* = 4), as well as Chinese databases such as China National Knowledge Infrastructure (CNKI) (*n* = 0) and VIP (*n* = 0). After deduplication, title/abstract screening, and full-text evaluation, 296 studies that did not meet the inclusion criteria were excluded, leaving a final sample of 54 eligible studies (Figure 1). All included studies evaluated video samples obtained from YouTube. Across the included studies, these video samples comprised a total of 5,051 individual videos, with a median video sample size of 83 individual videos per study (Figure 1).

**Figure 1:**
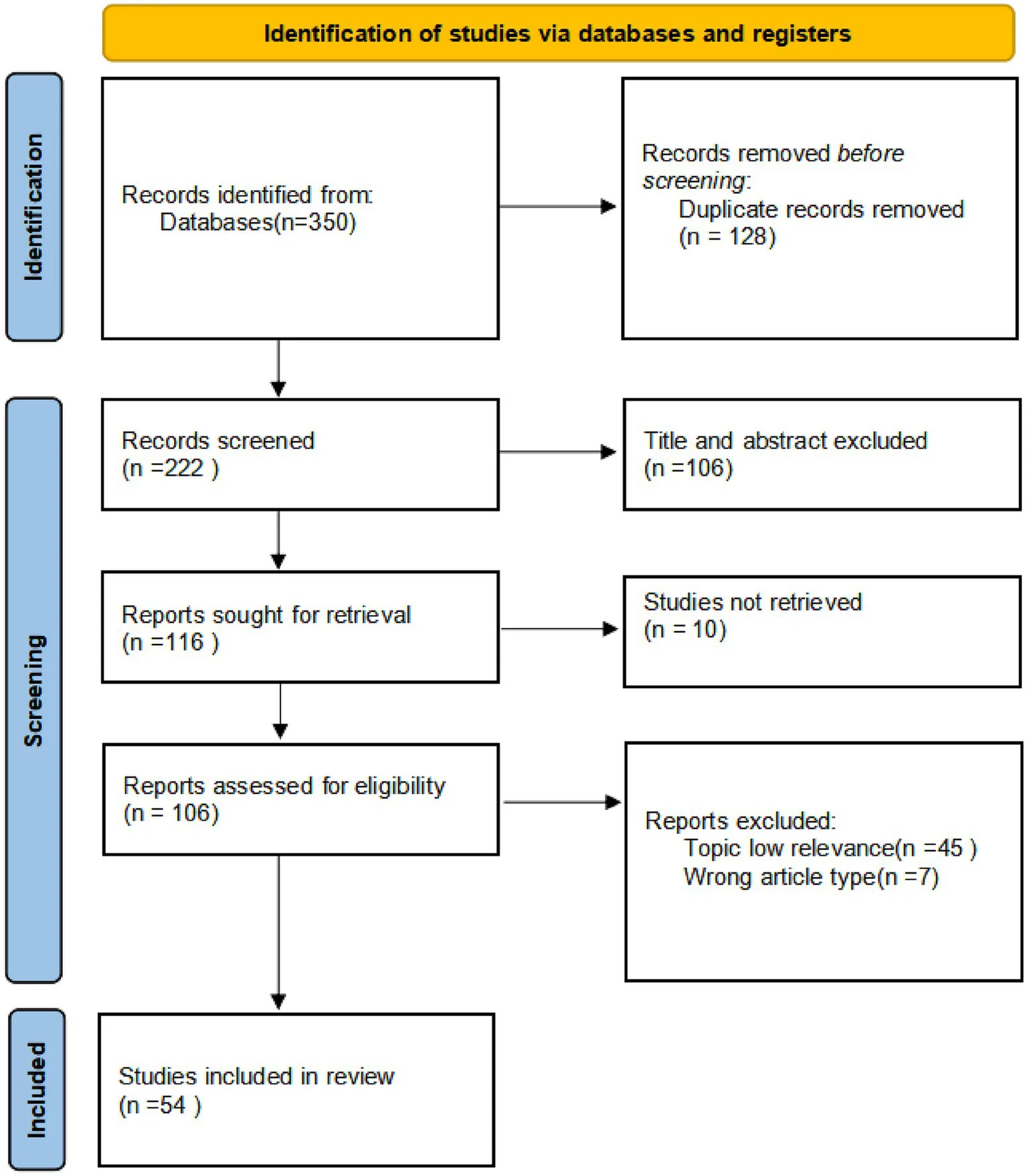
Flow diagram of study identification, screening, eligibility assessment, and inclusion according to the Preferred Reporting Items for Systematic Reviews and Meta-Analyses extension for Scoping Reviews (PRISMA-ScR); of 350 records identified, 54 studies were included.

### 3.2 Overview of cancer types and topic distribution

In terms of disease distribution, breast cancer (9 studies), prostate cancer (6 studies), and pan-cancer/general topics (5 studies) were the most frequently investigated categories (Figure 2). Based on research topics, the studies can be divided into 7 categories. Among these, the “Disease Information/General Introduction” category had the highest number of studies, with 23 (42.6%), primarily covering basic knowledge, epidemiological characteristics, and disease overviews of various cancers; followed by the “Screening/Early Detection/Self-Examination” category, with 10 studies (18.5%), focusing on breast self-examination, colorectal cancer screening, and HPV screening for cervical cancer; the “Treatment/Surgery/Procedures” category comprised 9 studies (16.7%), covering chemotherapy, radiation therapy, immunotherapy, and various surgical procedures; the “Rehabilitation/Supportive Care” category comprised 6 studies (11.1%), including cancer rehabilitation exercises, postoperative urinary incontinence management, and lymphedema care; the “Prevention/Vaccination/Risk Reduction” category comprised 3 studies (5.6%), primarily related to HPV vaccination; The “Diet/Nutrition” category comprised 2 studies (3.7%), focusing on nutritional management for patients with cancer and “real food” therapy; the “Diagnosis/Examinations” category was the smallest, comprising 1 study (1.9%) on procedures such as prostate biopsy (Figure 3). This distribution indicates that current studies evaluating the quality of cancer-related YouTube videos still focus primarily on disseminating basic disease knowledge and promoting early screening, while topics such as treatment, rehabilitation, and prevention receive relatively less attention (Figures 2, 3). These proportions reflect study-level topic distribution; video-level synthesis was not possible because comparable individual-video data were not consistently reported.

**Figure 2:**
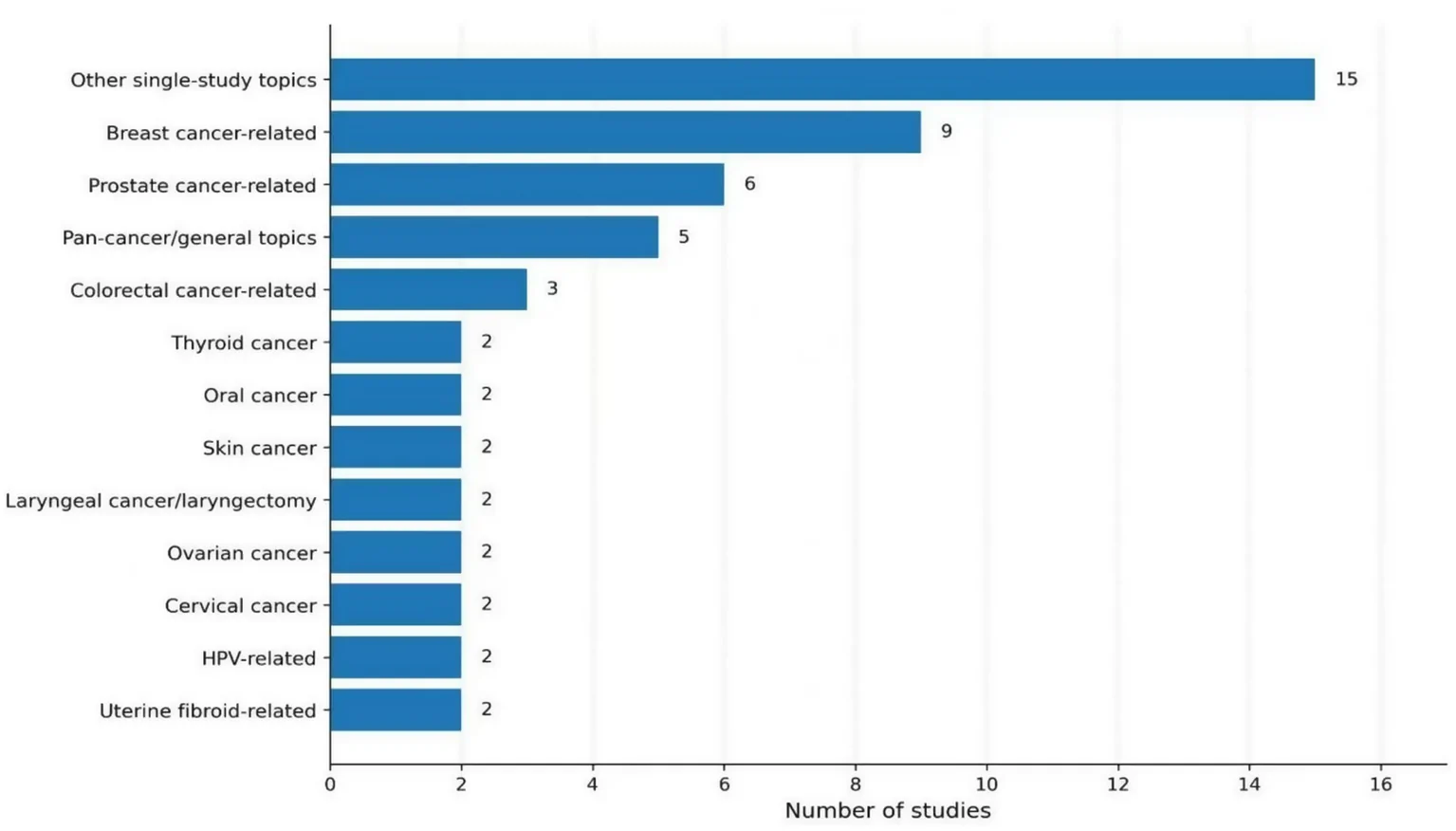
Distribution of cancer- and tumor-related topics across 54 included studies. Screening, treatment, rehabilitation, and postoperative care topics were grouped under the corresponding cancer or tumor type; categories represented by a single study were combined as “other single-study topics.” HPV, human papillomavirus.

**Figure 3:**
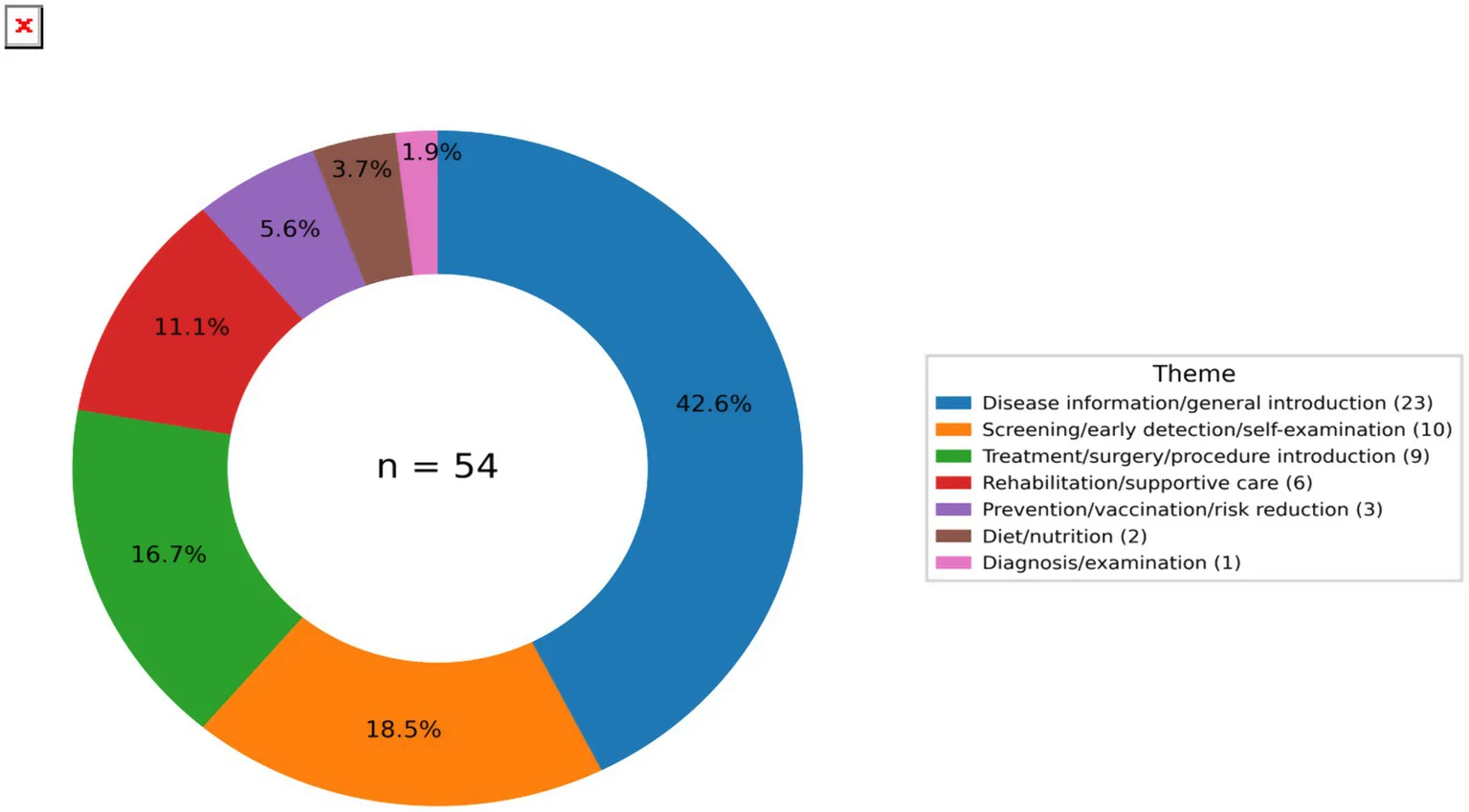
Distribution of primary content themes across the 54 included studies. Each study was assigned to one primary content theme according to its principal focus. Percentages may not sum to 100% because of rounding.

### 3.3 Video quality features and their evolution

Of the 54 included studies, 49 explicitly reported the date of the video search and were included in the descriptive temporal comparison: 5 were from the early period (2018–2019), 28 from the middle period (2020–2022), and 16 from the recent period (2023–2025); the remaining 5 were excluded from this comparison because they did not report the search date.

#### 3.3.1 Early phase (2018–2019)

A total of 5 studies were included, covering thyroid cancer (24), gastric cancer (25), breast cancer (26, 27), and prostate cancer (28), among others. All studies reported low or poor video quality. For example, the median DISCERN scores for thyroid cancer videos were 19.5 (range, 4–71) for reviewer 1 and 20.0 (range, 4–72) for reviewer 2; the JAMA score for laparoscopic gastrectomy videos was 1.94 out of 4; and 66% of breast cancer videos were rated “very poor.” No studies in this phase reported moderate or high quality.

#### 3.3.2 Mid-term (2020–2022)

A total of 28 studies covering prostate cancer (29), pancreatic cancer (30), testicular cancer (31), bladder cancer (32), colorectal cancer screening (33, 34), gynecologic cancers (35), hepatocellular carcinoma (36), and others. The quality assessment showed that most studies reported low- to moderate-quality video samples, with a few reporting high-quality video samples. Specifically, approximately 60% of the studies (17) reported low- or poor-quality video samples, such as those on prostate cancer screening, testicular cancer, and bladder cancer; approximately 35% of the studies (10) reported moderate-quality video samples, such as those on pancreatic cancer, oral self-examination (37), and hepatocellular carcinoma; only one study (postoperative exercise for breast cancer) (38) reported a high-quality video sample. It is worth noting that many of the moderate-quality videos were uploaded by professional medical institutions or physicians, whereas videos uploaded by individual patients were generally of lower quality.

#### 3.3.3 Recent (2023–2025)

A total of 16 studies, primarily covering lung cancer rehabilitation (39), ovarian cancer (40), laryngeal cancer (41), cervical cancer (42), vulvar cancer (43), oral cancer (44), and pediatric cancers (45), among others. There was a descriptive increase in the number of studies reporting moderate- or high-quality video samples: five studies reported high-quality video samples involving topics such as laryngeal cancer, cervical cancer (46), postoperative urinary incontinence following cancer surgery (47), and dental care for patients with head and neck cancer (48); ten reported moderate-quality video samples; and one reported a low-quality video sample involving ovarian cancer HIPEC videos (40). In particular, studies from 2024 to 2025 generally reported median GQS scores of 3 or 4 points (40, 42, 46, 49), with PEMAT comprehensibility scores exceeding 80%. Additionally, the proportion of videos uploaded by healthcare institutions increased, while commercial bias decreased (44, 49, 50) (Supplementary Table 3).

### 3.4 Use of evaluation tools

Among the 54 included cancer-related studies, the most frequently used evaluation tool was the GQS (Global Quality Score, 35 times), followed by DISCERN (27 times for the original version and 21 times for the modified version) and the JAMA tool (18 times); PEMAT (15 times) was also used frequently. In addition, tools such as VIQI (6 times), VPI (4 times), and HONcode (2 times) were occasionally used, while tools including MICI, MQ-VET, LIDA, FRES, and the Misinformation Scale each appeared only once (Figure 4). Overall, 50 studies (approximately 92.6%) used at least two evaluation tools, while only four studies used a single tool. The vast majority of studies employed a combination of 2 to 4 tools for multidimensional assessment, with some studies even using five or more (e.g., Reference (51) used five tools: mDISCERN, JAMA, GQS, VIQI, and HONcode; Reference (52) used five tools: PEMAT, JAMA, VPI, VIQI, and GQS) (Supplementary Table S2 and Figure 4).

**Figure 4:**
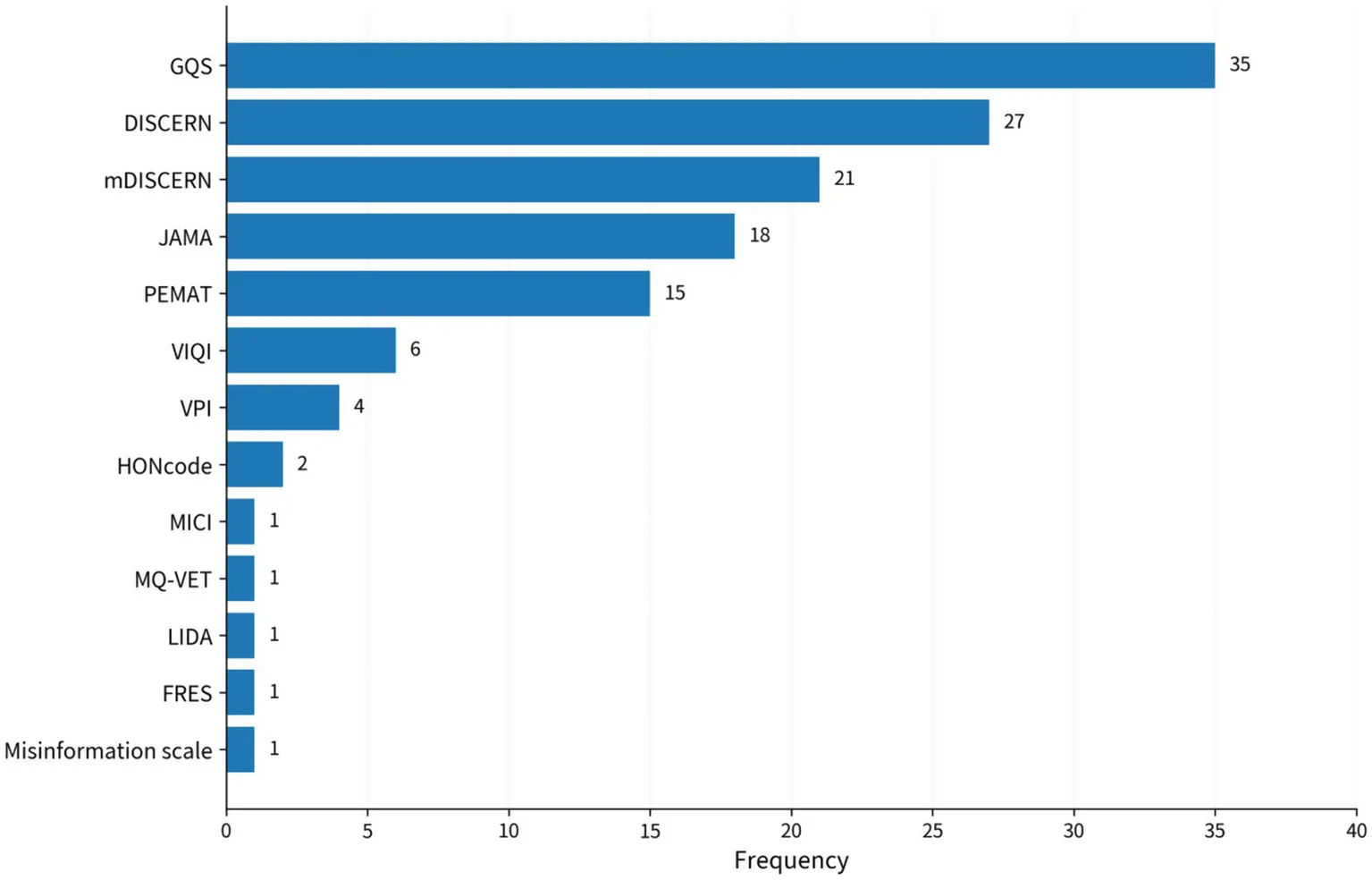
Frequency of assessment tools used in the included studies. Studies could use multiple tools; therefore, the total frequency exceeds 54. Original and modified DISCERN were counted separately. Abbreviations: DISCERN, an instrument for assessing the quality of consumer health information on treatment choices; FRES, Flesch Reading Ease Score; GQS, Global Quality Score; HONcode, Health On the Net Foundation Code of Conduct; JAMA, Journal of the American Medical Association benchmark criteria; LIDA, an instrument assessing the accessibility, usability, and reliability of online health information; mDISCERN, modified DISCERN; MICI, Medical Information and Content Index; MQ-VET, Medical Quality Video Evaluation Tool; PEMAT, Patient Education Materials Assessment Tool; VIQI, Video Information and Quality Index; VPI, Video Power Index.

Among commonly used tool combinations, GQS combined with DISCERN/mDISCERN is the most classic approach, balancing overall practicality with information reliability, though it lacks an assessment of patient comprehensibility and actionable content; adding PEMAT creates a three-dimensional coverage that comprehensively reflects the educational value of videos, but increases the time required for evaluation; DISCERN combined with JAMA focuses on academic credibility, making it suitable for screening highly rigorous resources, but it overlooks user-friendliness; Composite combinations including VIQI, VPI, and HONcode can be extended to cover information flow, popularity, and ethical transparency, making them suitable for cross-platform comparisons; however, they involve complex criteria and high implementation costs. Single tools are easy to use and offer high consistency, but their limited information dimensions make it difficult to comprehensively reflect video quality. The choice among these combinations should be based on a balance between research objectives and available resources.

The Global Quality Score (GQS), developed by Bernard et al. (54), is a single global five-point rating used to assess the overall information quality, flow, and usefulness of a video, rather than an instrument composed of five separate items. Scores range from 1 to 5, with higher scores indicating better overall quality and usefulness. Most included studies further categorized scores of 1–2, 3, and 4–5 as low, moderate, and high quality, respectively (53, 54).

The DISCERN instrument consists of 16 questions. Questions 1–8 assess the reliability of the publication, questions 9–15 assess the quality of information on treatment choices, and question 16 provides an overall quality rating. Questions 1–15 are each scored from 1 to 5, producing a total score ranging from 15 to 75. Total scores are commonly classified as very poor (15–26), poor (27–38), fair (39–50), good (51–62), or excellent (63–75). Question 16 provides a separate overall rating and should not be included in the 15-item total score. The original DISCERN and modified DISCERN instruments were recorded separately in this review (55–57).

The Modified DISCERN (mDISCERN) was adapted from the DISCERN tool and is more suitable for assessing video material. It consists of five questions related to the reliability of the video. Each question is scored 1 for “yes” and 0 for “no.” Higher scores correspond to higher reliability (53, 58).

JAMA Evaluation Criteria In 1997, the Journal of the American Medical Association (JAMA) proposed using the JAMA criteria to assess the transparency and accountability of online health information (59). These criteria consist of four standards: (1) authorship (video authors, affiliations, and credentials); (2) attribution (references, copyright information); (3) disclosure (sponsorship, advertising, commercial support, or information regarding potential conflicts of interest); (4) currency (dates of publication and updates). One point is awarded for each criterion met, with a score range of 0–4. A higher score indicates more complete reporting of transparency-related information, but does not directly establish the scientific accuracy of the video content (14, 59, 60).

The Patient Education Materials Assessment Tool (PEMAT) was developed in 2014 by the U. S. Agency for Healthcare Research and Quality (AHRQ) to assess the comprehensibility and implementability of health information (57). It includes two versions: one for evaluating printed materials (PEMAT-P) and one for evaluating video materials (PEMAT-A/V). PEMAT-A/V is an evaluation tool specifically designed for videos, with its items initially formulated to fully account for the characteristics of video media (61). PEMAT-A/V comprises 17 items: (1) Comprehensibility consists of 13 items, divided into five dimensions: “Content,” “Word Choice and Form,” “Organization,” “Layout Design,” and “Use of Visual Aids”; (2) Actionability consists of 4 items, with each item using a 3-point rating scale [i.e., Agree (1 point), Disagree (0 points), and Not Applicable (N/A)]. When it is not possible to make a determination based on the scoring criteria for a specific item, the “Not Applicable” (N/A) marker may be used. The overall score = [sum of scores for all items / (total number of items – number of items marked N/A)] × 100%, where the “sum of scores for all items” excludes items marked N/A (61, 62).

The VIQI tool encompasses four dimensions: information flow (VIQI 1), information accuracy (VIQI 2), quality (videos including one point for each image, animation, interview, video captions, and summary) (VIQI 3), and precision (level of coherence between video title and content) (VIQI 4). Each criterion is rated on a scale of 1 to 5, with higher scores indicating better quality (63, 64).

The Video Power Index (VPI), a metric developed by Erdem and widely employed in previous research, is used to indicate the popularity level of videos. The like ratio was calculated as (number of likes ×100)/(number of likes + number of dislikes), the view rate as (number of views/number of days since upload), and the VPI value, used to assess video popularity, as (like ratio × view rate)/100. After YouTube stopped publicly displaying dislike counts in November 2021, the original dislike-based like ratio and VPI could no longer be calculated from publicly available data. Therefore, this review extracted VPI values as reported by the included studies and did not impute missing dislike counts or recalculate VPI (65, 66).

The Health on the Net Code of Ethics (HONcode) was developed by the Health on the Net Foundation (HON), a non-governmental organization affiliated with the United Nations, with the aim of improving the reliability and comprehensibility of online health information. In 1996, HON published the HONcode on its website (17). The HONcode was one of the earliest tools for evaluating the quality of medical information on the Internet. Since then, researchers have applied it to evaluate video quality. The HONcode comprises eight criteria: authority, complementarity, privacy, attribution, reasonableness, transparency, disclosure of interests, and advertising policy. Based on the number of criteria met by a video—0–2, 3–5, or 6–8—videos are classified into three quality levels: low, moderate, and high, respectively (67–69).

### 3.5 Comparison of uploader types

Among the included studies that compared uploader types, most reported higher reliability, content comprehensiveness, or overall quality scores for videos uploaded by healthcare professionals, medical institutions, universities, or professional societies than for videos from non-professional or commercial uploaders. However, the measures and statistical comparisons varied across studies. For example, in prostate cancer (29, 50, 70–72), breast cancer (26, 40, 49, 73–77), pancreatic cancer (30), testicular cancer (31), bladder cancer (32), colorectal cancer (33, 34, 78), thyroid cancer (24, 79), ovarian cancer (40, 80), meningioma (81), and lung cancer rehabilitation (39), among others, consistently reported that videos from professional uploaders were of higher quality. Some studies further quantified these differences: for example, in studies on testicular cancer, all high-quality videos came from doctors or nonprofit organizations (*p* < 0.001); in studies on breast cancer, videos uploaded by legal entities were significantly higher in quality (*p* < 0.05); in studies on ovarian cancer, the reliability of videos from medical sources was significantly higher than that of non-medical sources (*p* = 0.001); in the pancreatic cancer study, videos uploaded by doctors had the highest quality and reliability. Conversely, videos uploaded by general-user uploaders (patients/individuals) tended to be of the lowest quality and were more likely to contain misleading information or commercial bias (e.g., 15–23% of prostate cancer screening videos contained commercial bias, mostly from for-profit companies). In summary, a broadly consistent pattern was observed across many cancer topics, with professionally produced videos generally receiving higher quality and reliability scores. (Supplementary Table S2).

### 3.6 The quality-popularity paradox

This review found a significant “quality–popularity paradox” in cancer-related videos on YouTube: high-quality, reliable professional videos tend to have low view counts, while low-quality, highly misleading personal narrative videos may actually be more popular (26, 32, 40, 45, 47, 75, 81, 82). For example, in a study of breast cancer videos (74), videos uploaded by legal entities were of significantly higher quality (median GQS of 3 vs. 2), but their median view count (163,454) and Video Popularity Index (VPI, 110) were both significantly lower than those of videos from blog sources (327,488 and 303), and quality scores were negatively correlated with daily view counts (*p* < 0.05); In the study on radioactive iodine therapy videos, the high-quality group had the lowest average view count (16,832), number of likes (83), and VPI (9.67), while the medium-quality group had the highest popularity (82); In the study of videos on nutrition for children with cancer, video quality scores were significantly negatively correlated with daily view rates (*r* = −0.359 to −0.404, *p* < 0.05), meaning that high-quality videos actually had lower view rates (45); However, the included studies did not directly evaluate YouTube’s recommendation algorithm; therefore, its role in disseminating lower-quality content remains unclear.

## 4 Discussion

### 4.1 The evolution, dissemination, and improvement of information quality

This study found that the overall quality of cancer- and tumor-related videos on YouTube was relatively low, consistent with the conclusions of a systematic review by Osman et al. (18) on the macro-level quality of health information on YouTube. However, a notable descriptive temporal difference is that, when grouped by the year in which the videos included in the studies were retrieved, the proportion of studies reporting high-quality video samples increased descriptively from 0% in 2018–2019 to 31.3% in 2023–2025. However, this result is based on the year the videos were retrieved for the included studies, rather than the upload date of each individual video; therefore, it reflects only a descriptive temporal difference at the study level and cannot be considered longitudinal evidence of a genuine improvement in the quality of YouTube cancer videos over time. This apparent improvement coincides temporally with the implementation of YouTube’s health information governance measures. In 2021, YouTube introduced source labels for health information and a dedicated health content section to help users identify videos from authoritative sources (84); in 2022, the platform further allowed eligible licensed healthcare professionals to apply for relevant health information features (85); in 2023, YouTube consolidated its medical misinformation policies into three categories—prevention, treatment, and denial—and explicitly removed content promoting harmful or ineffective cancer treatments, substituting standard treatments with unproven therapies, or discouraging patients from seeking professional medical help, while also creating authoritative cancer video playlists and collaborating with the Mayo Clinic to develop cancer content (86). While these measures may have created a more favorable environment for the dissemination of high-quality health content, official policy documents only confirm that the measures were implemented; they do not yet prove that they caused the temporal differences observed in this study.

Second, the involvement of healthcare professionals in content creation may also be related to this descriptive time discrepancy, as recent studies have shown an increase in the proportion of videos uploaded by healthcare institutions. At the same time, most studies comparing uploader types have found that cancer-related videos uploaded by doctors, hospitals, universities, or professional societies typically receive higher ratings for reliability, comprehensiveness, and overall quality. For example, a study of videos on prophylactic mastectomy found that videos uploaded by doctors were of good quality, those from health channels were of moderate quality, while videos uploaded by patients were of poor quality and misleading (49); in videos related to laryngeal cancer, videos by professional authors scored higher on both the GQS and the modified DISCERN scale than those by non-professional authors (41); in videos on vulvar cancer, those submitted by physicians were rated as relatively higher in quality according to the GQS (*p* = 0.045) (38). These studies support the notion that professional background may be associated with higher video quality. However, they did not directly examine whether the involvement of healthcare professionals has increased over time, nor can they prove that increased professional involvement has led to the rise in studies reporting higher-quality videos in recent years. Therefore, professional involvement can only serve as a possible explanation.

It should also be noted that the included studies differed in video retrieval and sampling procedures, languages, and assessment tools. Account and browser settings, geographic location, and the handling of YouTube Shorts may also have influenced the retrieved video samples, but these factors were insufficiently reported to allow systematic comparisons across studies. Therefore, differences in reported video quality may reflect not only the video content itself but also methodological and platform-related variation. Accordingly, comparisons across retrieval periods and cancer types should be interpreted descriptively rather than as direct or causal differences.

When examining the relationship between video quality and dissemination metrics, this review found a significant negative correlation between the quality and popularity of cancer-related videos on YouTube. Although personal experiences shared by ordinary users (patients, individual bloggers) receive lower information quality scores, they often authentically depict the struggles, courage, and daily realities of living with the disease. Their narratives may be highly compelling and evoke emotional resonance, which may be related to their high view counts and likes (47). However, since most of the included studies did not directly measure emotional resonance, this explanation remains speculative and cannot be regarded as a proven communication mechanism. In contrast, high-quality videos from professional uploaders, while accurate and comprehensive in content, tend to be presented in a more traditional manner and lack emotional engagement, which limits their dissemination effectiveness. This phenomenon suggests that, while ensuring the accuracy of professional information, science communication creators might draw on the techniques of narrative medicine (87) to integrate disease knowledge into storytelling. They should focus on a specific, detailed point for in-depth explanation rather than attempting to cover everything superficially, with the aim of simultaneously improving both content quality and dissemination effectiveness. Furthermore, platforms could explore validated quality indicators, expert verification processes, and clearer identification of professional medical sources when ranking or labeling health-related content.

Beyond the dual optimization of platform governance and expert involvement mentioned above, the critical discernment skills of viewers themselves must not be overlooked. This review found that while videos uploaded by ordinary users tend to evoke emotional resonance and attract high view counts, they often contain low-quality information and may even include explicitly misleading content or commercial biases (42, 77, 83). Therefore, when watching cancer-related videos on YouTube, viewers should proactively verify the source of the video (giving priority to accounts from hospitals, universities, professional societies, or certified physicians), be wary of subjective biases in personal experience-sharing, and exercise caution regarding content lacking evidence-based support, such as claims of “quick cures” or “alternative therapies.” Health literacy education should be an integral part of cancer health communication, helping patients and the public effectively identify reliable resources in an information-overloaded environment and avoid treatment delays or psychological harm resulting from believing false information.

### 4.2 Diversity of evaluation tools, trends toward multi-tool combinations, and challenges in standardization

This review found that the tools used in the YouTube Cancer Video Quality Evaluation Study exhibited a diverse landscape, with GQS (35 instances), original DISCERN (27 instances), modified DISCERN (21 instances), the JAMA Score (18 instances), and PEMAT (15 instances) being the most commonly used. This is largely consistent with the findings of a systematic review by Pavuloori et al. (19) on evaluation tools for YouTube surgical videos, indicating that DISCERN and GQS have become “core tools” in this field. Furthermore, over 92% of the studies employed a combination of two or more tools, far exceeding the approximately 50% multi-tool usage rate reported by Pavuloori et al. (19), reflecting the growing maturity of quality assessment methods in this field.

However, researchers should not blindly emulate existing multi-tool combinations but should carefully select tools whose evaluation dimensions align with the video’s core content themes. Taking the most commonly used the original DISCERN instrument as an example, this scale was originally designed to evaluate written health information regarding treatment option selection; its 16 items primarily focus on dimensions such as the reliability of treatment options, risks and benefits, and shared decision-making (13). Therefore, when evaluating cancer videos on therapeutic topics, such as the selection of radiotherapy and chemotherapy regimens or decisions regarding targeted drug therapy, the original DISCERN instrument demonstrates strong evaluative efficacy. However, when research focuses on cancer videos covering non-therapeutic topics, the original DISCERN instrument’s applicability is significantly diminished. For example, in lung cancer screening videos or educational videos introducing the definition, pathological classification, or risk factors of lung cancer the content aims to disseminate basic knowledge rather than assist patients in making treatment decisions. For such videos, most of the original DISCERN instrument’s items are inapplicable, leading to the possibility that even if the content is accurate and clearly presented, the video may receive a low score due to a mismatch with the scale’s dimensions, thereby introducing systematic bias. A combination of multiple tools can compensate to some extent for the shortcomings of a single tool; however, if the video content itself does not align with the assessment dimensions of any of the tools, increasing the number of tools will not resolve the fundamental issue. Therefore, researchers should first clarify the core theme of the video (treatment decision-making, basic knowledge, screening and prevention, etc.) and then select tools with matching dimensions, rather than mechanically applying commonly used combinations.

It is worth noting that, in addition to standardized scales, some studies have developed additional evaluation systems based on specific research questions, clinical guidelines, or self-developed scoring forms [e.g., the LAM content score in Yurdaisik (26) and the MICI scale in Duran and Kizilkan (31)]. Such customized tools can more precisely address the core concerns of specific cancer types or research topics, compensating for the shortcomings of general-purpose scales in detailed assessment, and thus possess certain innovative value and methodological significance. However, the reliability and validity of self-developed tools are often not systematically validated; they lack comparability across studies and may suffer from issues such as item bias or high rater subjectivity. Therefore, if such tools are used as the primary basis for evaluation, the robustness of the conclusions is open to question. It is recommended that researchers treat these tools as supplementary evaluation indicators, cross-validating them with standardized scales (such as GQS or DISCERN); alternatively, they should clearly document the development process, scoring criteria, and results of reliability and validity tests in their reports to enhance the credibility of the findings. Future research should also develop topic-specific or modular evaluation tools that allow for flexible selection of applicable dimensions based on content and incorporate patients and the general public as evaluators (38), in order to achieve more precise and equitable video quality assessments.

### 4.3 Research gaps and future directions

Currently, the vast majority of studies on video quality evaluation tend to take a diagnostic approach, highlighting issues such as “low video quality” and “unreliability,” as well as the active participation of healthcare professionals in science communication; however, few propose targeted improvement strategies based on actual data. Yet, as online health information has become an indispensable channel for the public to access medical knowledge, merely criticizing the current situation has proven somewhat ineffective. Future research may need to shift its perspective from “problem identification” to “strategy extraction”—that is, focusing on videos with outstanding dissemination outcomes (those receiving a large number of likes, comments, shares, and bookmarks) and conducting an in-depth analysis of the common strategies underlying their success. For example, do such videos incorporate narrative medicine techniques to enhance emotional resonance through real patient stories or clinicians’ clinical narratives? At what video length—typically within a few minutes—can optimal completion rates and information retention be achieved? What replicable best practices exist in terms of language style, visual presentation, and interactive guidance? By systematically comparing the characteristics of “high-reach” and “low-reach” videos, researchers can provide evidence-based creation guidelines for science communication creators, thereby enhancing dissemination effectiveness while ensuring information accuracy—and truly achieving “wide dissemination of high-quality content.” Furthermore, future research should develop topic-specific or modular evaluation tools tailored to different content themes such as screening, diagnosis, treatment, and rehabilitation; expand coverage of cancer types and topics, strengthening quality evaluations for videos on high-incidence cancers and prevention; and adopt longitudinal and intervention study designs to track changes in video quality and assess the effectiveness of professional interventions. Only by shifting from being “critics” to “builders” can this field provide a practical path toward improving the public health information environment.

### 4.4 Limitations

This review has several limitations. The included studies varied in cancer and tumor topics, video languages, search terms and dates, sampling methods and sample sizes, assessment tools and cut-off values, and engagement metrics; browser settings, geographic location, and the handling of YouTube Shorts were also incompletely reported, limiting direct comparisons across studies. Most studies were cross-sectional, and the same or overlapping videos may have been included in multiple studies; therefore, cumulative sample sizes should be interpreted as video observations rather than counts of unique videos. Temporal groups were based on study-level retrieval years rather than individual video upload dates, so the observed differences are descriptive and do not constitute longitudinal evidence of improvement in video quality. Because the assessment tools and scoring criteria differed, quality scores were not statistically pooled; future studies should use standardized retrieval, sampling, and evaluation methods in repeated cross-sectional or longitudinal designs.

## 5 Conclusion

This scoping review found that Cancer- and Tumor-related videos on YouTube were generally rated as low to moderate quality, but results varied across different cancer topics and evaluation methods. Due to the limited number of studies on some topics, it was not possible to make definitive comparisons for specific cancer types. Most studies comparing uploader types reported that videos produced by healthcare professionals or healthcare institutions received higher quality scores. In more recent studies, the proportion of research reporting samples of moderate- or high-quality videos was relatively higher in descriptive comparisons; at the same time, several studies reported that low-quality videos received higher levels of engagement than high-quality videos from professional sources. Given the significant methodological heterogeneity among the studies, both of these findings should be interpreted with caution. GQS, the original DISCERN, the modified DISCERN, and JAMA were the most commonly used evaluation tools, and most studies employed more than one evaluation tool. Strengthening the involvement of healthcare professionals and more clearly identifying and promoting credible health information may help enhance the educational value of cancer-related videos; at the same time, future studies need to adopt more standardized methods to enhance the reliability of the relevant evidence.

## Data Availability

The original contributions presented in the study are included in the article/Supplementary material, further inquiries can be directed to the corresponding author.

## References

[1] Sung H Filho AM Laversanne M Ferlay J Siegel RL Soerjomataram I . Global cancer statistics 2024: GLOBOCAN estimates of incidence and mortality worldwide for 34 cancers in 186 countries. CA Cancer J Clin. (2026) 76:e70090. doi: 10.3322/caac.70090, 42417444 PMC13343830

[2] Wörns MA Burns D Paskow M Makin H Miller J Turner LM . Patient experience of hepatocellular carcinoma and their treatment goals: an international qualitative study and patient journey map. Adv Ther. (2024) 41:3598–614. doi: 10.1007/s12325-024-02939-1, 39052152 PMC11349831

[3] Jiang XH Yuan XH Zhao H Peng JS. The effectiveness and feasibility of conversational agents in supporting Care for Patients with Cancer: systematic review and Meta-analysis. J Med Internet Res. (2025) 27:e76968. doi: 10.2196/76968, 40736462 PMC12374140

[4] Ludwigson A Huynh V Vemuru S Romandetti K Fisher C Coons HL . Characterizing informational needs and information seeking behavior of patients with breast cancer. Am J Surg. (2024) 227:100–5. doi: 10.1016/j.amjsurg.2023.09.047, 37863795 PMC11376347

[5] Lau N Srinakarin K Hong SJ Aalfs H Roth ME Ingram KM . Mobile health app attitudes and adoption among oncology providers: cross-sectional national survey. J Med Internet Res. (2026) 28:e85583. doi: 10.2196/85583, 41871339 PMC13054221

[6] Yao Y Zhang M Peng S Cheng Z Duan Y. Factors influencing continuance intention for online consultations among survivors of Cancer: grounded theory study. J Med Internet Res. (2026) 28:e84644. doi: 10.2196/84644, 41432715 PMC12824571

[7] Xu Z Jia J Wu C. Associations and pathways between online health information-seeking behavior and patient adherence: cross-sectional study. J Med Internet Res. (2026) 28:e91115. doi: 10.2196/91115, 42241710 PMC13235974

[8] Swire-Thompson B Lazer D. Public health and online misinformation: challenges and recommendations. Annu Rev Public Health. (2020) 41:433–51. doi: 10.1146/annurev-publhealth-040119-094127 31874069

[9] Zhang D Hu H Shi Z Li B. Perceived needs versus predisposing/enabling characteristics in relation to internet Cancer information seeking among the US and Chinese public: comparative survey research. J Med Internet Res. (2021) 23:e24733. doi: 10.2196/24733, 33427668 PMC7834927

[10] Loeb S Langford AT Bragg MA Sherman R Chan JM. Cancer misinformation on social media. CA Cancer J Clin. (2024) 74:453–64. doi: 10.3322/caac.21857, 38896503 PMC11648589

[11] Merl NB Schramm F Wies C Winterstein JT Brinker TJ. Generative AI in social media health communication: systematic review and meta-analysis of user engagement with implications for cancer prevention. Eur J Cancer. (2026) 232:116114. doi: 10.1016/j.ejca.2025.116114, 41252814

[12] Nguyen L Walters J Paul S Monreal Ijurco S Rainey GE Parekh N . Feeds, feelings, and focus: a systematic review and meta-analysis examining the cognitive and mental health correlates of short-form video use. Psychol Bull. (2025) 151:1125–46. doi: 10.1037/bul0000498, 41231585

[13] Zorlular A Zorlular R. YouTube as a tool for core stability exercise training a content evaluation study. BMC Public Health. (2026) 26:1662. doi: 10.1186/s12889-026-27323-2, 41975364 PMC13195914

[14] Liu XJ Valdez D Parker MA Mai A Walsh-Buhi ER. Quality of Cancer-related information on new media (2014-2023): systematic review and Meta-analysis. J Med Internet Res. (2025) 27:e73185. doi: 10.2196/73185, 41061257 PMC12547337

[15] Lim MSC Molenaar A Brennan L Reid M McCaffrey T. Young adults' use of different social media platforms for health information: insights from web-based conversations. J Med Internet Res. (2022) 24:e23656. doi: 10.2196/23656, 35040796 PMC8808344

[16] Lookingbill V Mohammadi E Cai Y. Assessment of accuracy, user engagement, and themes of eating disorder content in social media short videos. JAMA Netw Open. (2023) 6:e238897. doi: 10.1001/jamanetworkopen.2023.8897, 37074713 PMC10116364

[17] Song S Zhang Y Yu B. Interventions to support consumer evaluation of online health information credibility: a scoping review. Int J Med Inform. (2021) 145:104321. doi: 10.1016/j.ijmedinf.2020.104321 33202372

[18] Osman W Mohamed F Elhassan M Shoufan A. Is YouTube a reliable source of health-related information? A systematic review. BMC Med Educ. (2022) 22:382. doi: 10.1186/s12909-022-03446-z, 35590410 PMC9117585

[19] Pavuloori M Lin A Mi M. Tools/instruments for assessing YouTube videos on surgical procedures for patient/consumer health education: a systematic review. Front Public Health. (2025) 13:1575801. doi: 10.3389/fpubh.2025.1575801, 40709049 PMC12286933

[20] Tezcan H Akyildiz Tezcan E. Assessing the quality and reliability of cardiac rehabilitation information on YouTube: a systematic evaluation. Cureus J Med Sci. (2024) 16:e62752. doi: 10.7759/cureus.62752, 38912080 PMC11191395

[21] Karataş L Utkan Karasu A Demirsoy N. Is YouTube a sufficient and reliable source to inform patients about cardiac rehabilitation?: a cross-sectional study. J Cardiopulm Rehabil Prev. (2024) 44:239–47. doi: 10.1097/HCR.0000000000000864, 38875164

[22] Kocyigit BF Nacitarhan V Koca TT Berk E. YouTube as a source of patient information for ankylosing spondylitis exercises. Clin Rheumatol. (2019) 38:1747–51. doi: 10.1007/s10067-018-04413-0, 30645752

[23] Assylbek MI Zimba O Akyol A Yessirkepov M Kocyigit BF. YouTube as a source of information for stroke rehabilitation: a cross-sectional analysis of quality and reliability of videos. Rheumatol Int. (2025) 45:77. doi: 10.1007/s00296-025-05832-4, 40119932 PMC11929695

[24] Aydin MA Akyol H. Quality of information available on YouTube videos pertaining to thyroid Cancer. J Cancer Educ. (2020) 35:599–605. doi: 10.1007/s13187-019-01502-9, 30838529

[25] Zhang S Fukunaga T Oka S Orita H Kaji S Yube Y . Concerns of quality, utility, and reliability of laparoscopic gastrectomy for gastric cancer in public video sharing platform. Ann Transl Med. (2020) 8:196. doi: 10.21037/atm.2020.01.78, 32309343 PMC7154475

[26] Yurdaisik I. Analysis of the most viewed first 50 videos on YouTube about breast cancer. Biomed Res Int. (2020) 2020:2750148. doi: 10.1155/2020/2750148, 32596288 PMC7273466

[27] Esen E Aslan M Sonbahar BÇ Kerimoğlu RS. YouTube English videos as a source of information on breast self-examination. Breast Cancer Res Treat. (2019) 173:629–35. doi: 10.1007/s10549-018-5044-z, 30443880

[28] Loeb S Sengupta S Butaney M Macaluso JN Jr Czarniecki SW Robbins R . Dissemination of misinformative and biased information about prostate cancer on YouTube. Eur Urol. (2019) 75:564–7. doi: 10.1016/j.eururo.2018.10.056, 30502104

[29] Shungu N Haley SP Berini CR Foster D Diaz VA. Quality of YouTube videos on prostate cancer screening for black men. J Am Board Fam Med. (2021) 34:724–31. doi: 10.3122/jabfm.2021.04.200632, 34312265

[30] Cakmak G Mantoglu B. Reliability and quality of YouTube contents pertaining to pancreatic cancer. Cureus. (2021) 13:e14085. doi: 10.7759/cureus.14085, 33907636 PMC8065091

[31] Duran MB Kizilkan Y. Quality analysis of testicular cancer videos on YouTube. Andrologia. (2021) 53:e14118. doi: 10.1111/and.14118, 34009641

[32] García-Cano-Fernández AM Szczesniewski-Dudzik JJ García-Tello A Diego-García V Boronat-Catalá J Llanes-González L. Quality of bladder cancer information on YouTube. Cent Eur J Urol. (2022) 75:248–51. doi: 10.5173/ceju.2022.151, 36381156 PMC9628723

[33] Yalkin O Uzunoglu MY İflazoglu N Esen E. Are YouTube videos a reliable source for obtaining information about colorectal Cancer screening? Cureus. (2022) 14:e21550. doi: 10.7759/cureus.21550, 35223321 PMC8865597

[34] Kahlam A Pai S Kahlam J Ahlawat S. Quality of YouTube videos related to colorectal cancer screening. Cureus. (2023) 15:e33684. doi: 10.7759/cureus.33684 PMC9920494 36788914

[35] Ekmez F Ekmez M. Evaluation of the quality and reliability of YouTube videos with Turkish content as an information source for gynecological cancers during the COVID-19 pandemic. Cureus. (2023) 15:e44581. doi: 10.7759/cureus.44581, 37662510 PMC10474934

[36] Kaplan K Solak Y. Evaluation of YouTube videos on hepatocellular carcinoma. J Korean Med Sci. (2023) 38:e50. doi: 10.3346/jkms.2023.38.e50, 36808545 PMC9941019

[37] Gulve ND Tripathi PR Dahivelkar SD Gulve MN Gulve RN Kolhe SJ. Evaluation of YouTube videos as a source of information about Oral self-examination to detect Oral Cancer and precancerous lesions. J Int Soc Prev Community Dent. (2022) 12:226–34. doi: 10.4103/jispcd.JISPCD_277_21, 35462738 PMC9022386

[38] Rodriguez Rodriguez AM Blanco-Díaz M Lopez Diaz P de la Fuente Costa M Dueñas L Escobio Prieto I . Quality analysis of YouTube videos presenting shoulder exercises after breast cancer surgery. Breast Care (Basel). (2022) 17:188–98. doi: 10.1159/000518265, 35747900 PMC9162479

[39] Kocamaz D Demircioğlu Karagöz A Atasavun Uysal S. YouTube videos as an information source about aerobic exercise in rehabilitation of lung cancer. Integr Cancer Ther. (2025) 24:15347354251331461. doi: 10.1177/15347354251331461 PMC12034951 40238487

[40] Mezzapesa F Bilancia EP Afonina M Di Costanzo S Masina E De Iaco P . Is YouTube™ a reliable source of information for the current use of HIPEC in the treatment of ovarian cancer? Cancers (Basel). (2025) 17:3222. doi: 10.3390/cancers17193222, 41097747 PMC12523930

[41] Liu Z Chen Y Lin Y Ai M Lian D Zhang Y . YouTube/ Bilibili/ TikTok videos as sources of medical information on laryngeal carcinoma: cross-sectional content analysis study. BMC Public Health. (2024) 24:1594. doi: 10.1186/s12889-024-19077-6, 38877432 PMC11177428

[42] Qi S Chen Q Liang J Yang X Li X. Evaluating the quality and reliability of cervical cancer related videos on YouTube, Bilibili, and Tiktok: a cross-sectional analysis. BMC Public Health. (2025) 25:3682. doi: 10.1186/s12889-025-24840-4, 41174620 PMC12577082

[43] Xu T Zhao H. Analysis of the quality of vulvar cancer-related videos on YouTube. Transl Cancer Res. (2025) 14:102–11. doi: 10.21037/tcr-24-1411, 39974393 PMC11833394

[44] Zhang Q Li Z Zhang H Han L Zhao S Jia S. YouTube and Bilibili as sources of information on oral cancer: cross-sectional content analysis study. Sci Rep. (2025) 15:21671. doi: 10.1038/s41598-025-02898-9, 40593960 PMC12214728

[45] Sözlü S Macit-Çelebi MS Kocaadam-Bozkurt B. YouTube videos on nutrition and pediatric cancer: scientific reliability and quality. Front Public Health. (2026) 14:1767637. doi: 10.3389/fpubh.2026.1767637, 41835415 PMC12982093

[46] Liu Y Wu R Zhang L. A cross-sectional study on the quality of cervical cancer health information across multiple short video platforms: analysis of content, quality, and dissemination characteristics. Digit Health. (2026) 12:20552076261415927. doi: 10.1177/20552076261415927, 41585303 PMC12830547

[47] Rodriguez-Rodriguez AM De la Fuente-Costa M Escalera-de la Riva M Domínguez-Navarro F Perez-Dominguez B Paseiro-Ares G . Assessing the quality of YouTube's incontinence information after Cancer surgery: an innovative graphical analysis. Healthcare (Basel). (2024) 12:243. doi: 10.3390/healthcare12020243, 38255130 PMC10815186

[48] Marschner F Runkel L Range M Lechte C Wiegand A. How reliable is eHealth information on dental care in head and neck cancer? A quality evaluation. BMC Oral Health. (2026) 26:814. doi: 10.1186/s12903-026-08488-z, 42050480 PMC13157662

[49] Altintas T Bilgi Kirmaci M. Evaluating YouTube videos with prophylactic mastectomy content in terms of their quality and reliability. Breast J. (2025) 2025:9487931. doi: 10.1155/tbj/9487931, 39830622 PMC11737905

[50] Sandıkcı F Altan M. Quality, reliability, and content analysis of YouTube videos on transperineal prostate biopsy. Digit Health. (2026) 12:20552076261432019. doi: 10.1177/20552076261432019, 41883531 PMC13009971

[51] Sahin E Seyyar M. Assessing the scientific quality and reliability of YouTube videos about chemotherapy. Medicine (Baltimore). (2023) 102:e35916. doi: 10.1097/MD.0000000000035916, 37960752 PMC10637493

[52] Özeller E Toçoğlu M Aktı-Çakır E Hüsna SÇ. Analyzing Turkish-language HPV vaccination videos on YouTube: assessing content quality and educational value. Infect Dis Clin Microbiol. (2025) 7:310–9. doi: 10.36519/idcm.2025.699, 41111497 PMC12532016

[53] Sun F Zheng S Wu J. Quality of information in gallstone disease videos on TikTok: cross-sectional study. J Med Internet Res. (2023) 25:e39162. doi: 10.2196/39162, 36753307 PMC9947761

[54] Bernard A Langille M Hughes S Rose C Leddin D Veldhuyzen van Zanten S . A systematic review of patient inflammatory bowel disease information resources on the World Wide Web. Am J Gastroenterol. (2007) 102:2070–7. doi: 10.1111/j.1572-0241.2007.01325.x 17511753

[55] Charnock D Shepperd S Needham G Gann R. DISCERN: an instrument for judging the quality of written consumer health information on treatment choices. J Epidemiol Community Health. (1999) 53:105–11. doi: 10.1136/jech.53.2.105, 10396471 PMC1756830

[56] Wu J Trahair E Happ M Swartz J. TikTok, #IUD, and user experience with intrauterine devices reported on social media. Obstet Gynecol. (2023) 141:215–7. doi: 10.1097/AOG.0000000000005027, 36473194 PMC9892286

[57] Zhang J He J Wei Q Rao M. Evaluation of the quality and reliability of infertility information on Chinese social media platforms Bilibili and TikTok: a cross-sectional study. BMC Public Health. (2026) 26:2639. doi: 10.1186/s12889-026-28306-z, 42387332 PMC13595763

[58] Tosun A Reyhanioglu DA. Content and quality analysis of YouTube videos on therapeutic exercises for Parkinson's disease. J Parkinsons Dis. (2026) 16:956–64. doi: 10.1177/1877718X261438620, 41983917 PMC13435159

[59] Silberg WM Lundberg GD Musacchio RA. Assessing, controlling, and assuring the quality of medical information on the internet: caveant lector et viewor--let the reader and viewer beware. JAMA. (1997) 277:1244–5. 9103351

[60] Zhou R Li Y Yan C Xu C Feng S. TikTok and Bilibili as sources of palliative care information: an analysis of quality and reliability. BMC Palliat Care. (2026) 25:103. doi: 10.1186/s12904-026-02051-x, 41820900 PMC13094118

[61] Shoemaker SJ Wolf MS Brach C. Development of the patient education materials assessment tool (PEMAT): a new measure of understandability and actionability for print and audiovisual patient information. Patient Educ Couns. (2014) 96:395–403. doi: 10.1016/j.pec.2014.05.027, 24973195 PMC5085258

[62] Li Y Ouyang H Lin G Peng Y Yao J Chen Y. Evaluation of the measurement properties of online health information quality assessment tools: a systematic review. Int J Nurs Sci. (2025) 12:130–6. doi: 10.1016/j.ijnss.2025.02.015, 40241869 PMC11997670

[63] Lian D Zhang Y Liu Z Lin Y Ai M Chen Y . Public health implications of allergic rhinitis information on YouTube and Bilibili: a cross-cultural analysis of content quality, engagement, and seasonal trends. BMC Public Health. (2026) 26:2375. doi: 10.1186/s12889-026-28015-7, 42271340 PMC13479579

[64] Yildirim G Kocaelli HA. Assessment of the content and quality of YouTube videos related zygomatic implants: a content-quality analysis. Clin Implant Dent Relat Res. (2023) 25:605–12. doi: 10.1111/cid.13194, 36808698

[65] Weitzner AS Simpkins A Yang J Martheswaran T Perrigo M Crigger CB. Bladder exstrophy on YouTube: an evaluation of quality, actionability, and user engagement. World J Urol. (2026) 44:297. doi: 10.1007/s00345-026-06229-z, 41964761 PMC13070057

[66] Li Z Yan C Lyu X Li F Zeng R. Assessing quality and reliability of online videos on tachycardia: a YouTube video-based study. BMC Public Health. (2024) 24:2620. doi: 10.1186/s12889-024-20062-2, 39334090 PMC11438393

[67] Health On The Net. About health on the net. Available online at: https://www.hon.ch/en/about.html (Accessed August 15, 2023).

[68] Treadgold BM Coulson NS Campbell JL Lambert J Pitchforth E. Quality and misinformation about health conditions in online peer support groups: scoping review. J Med Internet Res. (2025) 27:e71140. doi: 10.2196/71140, 40377972 PMC12125560

[69] Buschulte K El-Hadi S Höger P Ganter C Wijsenbeek M Kahn N . Is YouTube a sufficient source of information on sarcoidosis? Respir Res. (2024) 25:334. doi: 10.1186/s12931-024-02956-2, 39252025 PMC11386059

[70] Vu E Pratsinis M Plasswilm L Schmid HP Panje C Betschart P. Radiotherapy or surgery? Comparative, qualitative assessment of online patient education materials on prostate cancer. Curr Oncol. (2021) 28:3420–9. doi: 10.3390/curroncol28050296, 34590594 PMC8482178

[71] Abramson M Feiertag N Javidi D Babar M Loeb S Watts K. Accuracy of prostate cancer screening recommendations for high-risk populations on YouTube and TikTok. BJUI Compass. (2022) 4:206–13. doi: 10.1002/bco2.200, 36816146 PMC9931542

[72] Baqain L Mukherji D Al-Shamsi HO Abu-Gheida I Ibraheem AA Rabii KA . Quality and reliability of YouTube videos in Arabic as a source of patient information on prostate cancer. Ecancermedicalscience. (2023) 17:1573. doi: 10.3332/ecancer.2023.1573, 37533942 PMC10393303

[73] Ayoub G Chalhoub E Sleilaty G Kourie HR. YouTube as a source of information on breast cancer in the Arab world. Support Care Cancer. (2021) 29:8009–17. doi: 10.1007/s00520-021-06403-6, 34224018

[74] Morena N Ahisar Y Wang X Nguyen D Rentschler CA Meguerditchian AN. Content quality of YouTube videos about metastatic breast Cancer in young women: systematic assessment. JMIR Cancer. (2023) 9:e45101. doi: 10.2196/45101, 37737837 PMC10685279

[75] Bu Bshait MS Almaqhawi A. Quality assessment of YouTube videos as information source for breast self-examination. Cureus. (2024) 16:e70227. doi: 10.7759/cureus.70227, 39463531 PMC11512166

[76] Fernandez-Alvarez MDM Cachero-Rodríguez J Fu MR Sánchez-Fernández P Ureña-Lorenzo A Martin-Payo R. YouTube videos on lymphedema as an information source for Spanish speaking breast cancer survivors. Support Care Cancer. (2024) 32:540. doi: 10.1007/s00520-024-08746-2, 39044016 PMC11266410

[77] Soroudi D Falade I Mehta N Gursky A Yin R Song S . Evaluating the quality and reliability of oncologic breast reconstruction videos on youtube. JPRAS Open. (2024) 42:311–4. doi: 10.1016/j.jpra.2024.10.006, 39524906 PMC11549973

[78] Chen Z Pan S Zhou M Wang X. Evaluation of the quality and reliability of anal cancer and its precancerous lesions-related content on YouTube: a cross-sectional study. BMJ Open. (2023) 13:e074787. doi: 10.1136/bmjopen-2023-074787, 37852760 PMC10603535

[79] Kang E Ju H Kim S Choi J. Contents analysis of thyroid cancer-related information uploaded to YouTube by physicians in Korea: endorsing thyroid cancer screening, potentially leading to overdiagnosis. BMC Public Health. (2024) 24:942. doi: 10.1186/s12889-024-18403-2, 38566004 PMC10985908

[80] Jeong SY Kim J Hwang J Park ST Park SH. The role of YouTube as an information platform for ovarian cancer: the educational quality and reliability. Yonsei Med J. (2026) 67:502–7. doi: 10.3349/ymj.2025.0374, 42198861 PMC13213268

[81] Krakowiak M Fercho J Piwowska K Yuser R Szmuda T Słoniewski P. YouTube as a source of patient information for Meningiomas: a content quality and audience engagement analysis. Healthcare (Basel). (2022) 10:506. doi: 10.3390/healthcare10030506, 35326984 PMC8953264

[82] Korkmaz U Soyluoglu S Arda E. Radionuclide therapy videos on YouTube as an educational material: has the COVID-19 pandemic changed the quality, usefulness, and interaction features. Nucl Med Mol Imaging. (2023) 57:1–7. doi: 10.1007/s13139-023-00799-1 PMC10073796 37360576

[83] Segado-Fernández S Herrera-Peco I Jiménez-Gómez B Ruiz Núñez C Jiménez-Hidalgo PJ Benítez de Gracia E . Realfood and cancer: analysis of the reliability and quality of YouTube content. Int J Environ Res Public Health. (2023) 20:5046. doi: 10.3390/ijerph20065046, 36981954 PMC10048849

[84] Graham G. Introducing new ways to help you find answers to your health questions YouTube Official Blog (2021) Available online at: https://blog.youtube/news-and-events/introducing-new-ways-help-you-find-answers-your-health-questions/ (Accessed August 19, 2026).

[85] Graham G. New ways for licensed healthcare professionals to reach people on YouTube YouTube Official Blog (2022). Available online at: https://blog.youtube/news-and-events/new-ways-licensed-healthcare-professionals-product-feature-application/ (Accessed August 19, 2026).

[86] Graham G. Halprin M. A long term vision for YouTube’s medical misinformation policies YouTube Official Blog (2023). Available online at: https://blog.youtube/inside-youtube/a-long-term-vision-for-medical-misinformation-policies/ (Accessed August 19, 2026).

[87] Launer J Wohlmann A. Narrative medicine, narrative practice, and the creation of meaning. Lancet. (2023) 401:98–9. doi: 10.1016/S0140-6736(23)00017-X, 36641204

