# Supplementary material for: Quality and reliability of cancer- and tumor-related videos on YouTube: a scoping review
Source: Front Public Health. 2026 Oct 14;14:1940232. doi: 10.3389/fpubh.2026.1940232 (PMC13713483; doi:10.3389/fpubh.2026.1940232)
Supplement: Supplementary file 1 [file Supplementary_Table_3.docx]

**Table 1. Characteristics of included studies**

| **Included studies** | **Author** | **Topic** | **Data Collection Period** | **Sample size** | **Assessment Tools** | **Key Findings** |
| --- | --- | --- | --- | --- | --- | --- |
| 1 | Aydin MA  et al.[1]  2020 | Thyroid cancer | 2018/2/12 | 52 | 1、DISCERN 2、JAMA | 1. Median DISCERN score for videos: 19.5–20 points; median JAMA score: 2 points, with generally poor quality.  2. Video length is positively correlated with both DISCERN and JAMA scores.  3. JAMA scores are positively correlated with the video popularity index (r = 0.31–0.36, p < 0.05). |
| 2 | Esen E  et al.[2]  2019 | breast self-examination | 2018/6/21 | 87 | 1、GQS | 1. 62% of the videos contained misleading information, and their reliability, content, and quality scores were significantly lower than those of the useful information group (p < 0.001).  2. misleading videos were mostly uploaded by individual users, their view counts were higher than those of the useful information group (difference was not statistically significant). |
| 3 | Korkmaz U  et al.[3]  2023 | Radiation therapy | 2018/8/25 | 103 | 1、JAMA 2、GQS | 1. The usefulness rates of the videos were 87.3% and 97.9%, respectively, with high-quality videos (JAMA 4 points/GQS 4–5 points) predominating.  2. The popularity of video usefulness was negatively correlated with quality (r = −0.370, p = 0.001). |
| 4 | Rodriguez  et al.[4]  2022 | Exercise After Breast Cancer Surgery | 2019/3/31 | 51 | 1、VPI 2、DISCERN 3、GQS | 1. The video quality is relatively high (with an average DISCERN score of 50.97, an average GQS of 3.49, and 67% of the videos rated as high or very high). |
| 5 | Zhang S  et al.[5]  2020 | Laparoscopic Resection for Gastric Cancer | 2019/8/16 | 102 | 1、JAMA | 1. JAMA benchmark score of 1.94 (range: 1 to 4); video quality varies widely; due to insufficient comments, the information is quite incomplete. |
| 6 | Shungu N  et al.[6]  2021 | Prostate Cancer Screening | 2020 | 50 | 1、DISCERN 2、PEMAT | 1. The average DISCERN score (standard deviation) was 40.24 (12.49), and the average PEMAT score (standard deviation) was 9.31 (1.78).  2. There was a significant positive correlation between PEMAT scores and view counts for videos of lower overall quality (Kendall’s τ = 0.308, P = 0.002). |
| 7 | Wilkens FM[7]  et al.  2022 | lymphangioleiomyomatosis | 2020/4/20 | 64 | 1、DISCERN 2、HONcode | 1. Video quality was generally poor (median DISCERN score of 28/80, 53% of videos with a HONcode score of ≤2, median LAM content score of 8/31); only a few videos provided high-quality and reliable information. |
| 8 | Ayoub G  et al.[8]  2021 | breast cancer in the Arab world | 2020/5/20 | 60 | 1、mDISCERN 2、GQS | 1. The overall quality of the videos was poor (median GQS score of 3/5, reliability of 2/5, and content of 4/11). Forty-two percent of the videos contained misleading information; videos uploaded by legal entities were of significantly higher quality (p < 0.05) but were less popular.  2. GQS was negatively correlated with views per video (p < 0.05). |
| 9 | Steeb T  et al.[9]  2022 | Basal cell carcinoma | 2020/7/1 | 41 | 1、VPI  2、DISCERN 3、GQS | 1. Video quality was moderate to good (DISCERN 3.3 ± 0.80, GQS 3.8 ± 1.1), with high comprehensibility (70.8% ± 13.3%), but low actionability (45.9% ± 43.7%) and reliability (JAMA score 27.74% ± 22.1%). Furthermore, videos produced by healthcare professionals were of significantly higher quality than those produced by non-professionals. |
| 10 | Richardson MA  et al.[10]  2022 | Spinal Tumors | 2020/7/7 | 50 | 1、JAMA 2、GQS | 1. The videos had an average JAMA score of 3.1 ± 0.27 and an average GQS of 2.6 ± 1.3, indicating that their overall reliability and educational quality ranged from poor to suboptimal. |
| 11 | Cakmak G  et al.[11]  2021 | Pancreatic cancer | 2020/10/10 | 50 | 1、GQS 2、mDISCERN | 1. The average video quality score (GQS) was 3.21 ± 0.94, and the reliability score (DISCERN) was 3.47 ± 0.99, indicating an overall moderate level of quality; furthermore, only 6% of the videos were uploaded directly by physicians.  2. Videos uploaded by physicians had the highest quality and reliability. |
| 12 | Kahlam A  et al.[12]  2023 | Colorectal Cancer Screening | 2020/10/10 | 50 | 1、DISCERN | 1. The overall video quality is average (average DISCERN score of 3.16 out of 5) |
| 13 | Yalkin O  et al.[13]  2022 | Colorectal Cancer Screening | 2020/11/19 | 138 | 1、DISCERN 2、GQS | 1. 54% (74 out of 137) of the videos contained useful information, with 47.3% of these coming from academic sources, while videos containing misleading information (46%, 63 out of 137) mostly originated from for-profit companies.  2. Videos containing useful information received significantly more likes (p=0.013) and scored significantly higher in terms of reliability, comprehensiveness, and quality; however, the videos with the highest view counts—those that garnered the most public attention—actually had lower content quality. |
| 14 | Ekmez F  et al.[14]  2023 | Gynecological Cancers | 2020/12/2-5 | 87 | 1、mDISCERN 2、MICI 3、VIQI | 1. Patient experience videos had significantly higher view counts (p=0.001)  2. Informational videos had significantly higher DISCERN scores (3.2 vs. 1.6/0.8) and VIQI total scores (11.9 vs. 8.8/7.2) (p=0.001). Overall quality was moderate. Although patient experience videos were the most popular (average views of 3,329.7 ± 1,310.6) |
| 15 | Gad B  et al.[15]  2022 | Uterine Fibroid Embolization | 2021 | 31 | 1、DISCERN | 1. Quality is average or poor (average DISCERN score: 48.82 ± 14.48) |
| 16 | Duran MB  et al.[16]  2021 | Testicular cancer | 2021/2/15 | 152 | 1、JAMA 2、mDISCERN 3、GQS | 1. JAMA Score: Mean ± SD (1.59 ± 1.11); mDISCERN Tool: Mean ± SD (2.13 ± 1.02); Global Quality Score: Mean ± SD (2.61 ± 1.04).  2. Most video content on YouTube regarding testicular cancer is of low to moderate quality (only 9.9% is high quality).  3. All high-quality videos originate from doctors, nonprofit organizations, universities, or professional institutions  (p < 0.00). The average duration of high-quality videos (2,247 seconds) far exceeds that of low-quality videos (310 seconds), and duration is significantly positively correlated with all quality scores.（r=0.33~0.35, p<0.001） |
| 17 | Bahar-Ozdemir Y  et al.[17]  2021 | Cancer rehabilitation | 2021/2/26 | 53 | 1、mDISCERN 2、JAMA 3、GQS | 1. Modified DISCERN (mean ± standard deviation) = 2.14 ± 1.0, JAMA (mean ± standard deviation) = 2.03 ± 1.01, GQS scores (mean ± standard deviation) = 2.78 ± 0.89. Cancer recovery videos on YouTube are of low quality, and the information provided is insufficient and unreliable.  2. Videos provided by physical therapists were of the highest quality. The videos were of low quality, accounting for only about one-seventh (14.4%) of the total. |
| 18 | Vu E  et al.[18]  2021 | Prostate Cancer Treatment | 2021/3/1 | 80 | 1、DISCERN 2、PEMAT | 1. DISCERN median [range] 43 points (20.5–65); chemotherapy DISCERN median [range] 36.5 points (18–71); radiotherapy PEMAT median interpretability score 60% (range 0–100%); surgery median 75% (range 40–100). Most of the provided video content is of insufficient quality and may be subject to commercial bias.  2. Videos posted by healthcare professionals (doctors, clinics, hospitals, universities) were of the highest quality (U = 67.5, p < 0.05) |
| 19 | García-Cano-Fernández AM  et al.[19]  2022 | Bladder cancer | 2021/3/9 | 38 | 1、PEMAT 2、DISCERN | 1. Over 60% of the videos were of low quality, with a median PEMAT comprehensibility score of 71.6%, a median PEMAT actionable score of 35.5%, and a median DISCERN total score of 34; 66.7% of the videos were of low quality.  2. Videos featuring healthcare professionals as the main characters: 90.9% were of low quality (20 out of 22), while only 9.1% were of medium or high quality. |
| 20 | Krakowiak M  et al.[20]  2022 | Meningiomas | 2021/4/10 | 119 | 1、DISCERN 2、GQS 3、JAMA | 1、DISCERN score median of 33.8 (±9.2)，GQS scale, the mean score was 2.4 (range 1–4)，JAMA, the mean score was 1.8 (range 1–4), the median 1.5。 2. DISCERN scores were positively correlated with video duration (p < 0.05, r = 0.4) |
| 21 | Morena N  et al.[21]  2023 | Metastatic Breast Cancer | 2021/8/3 | 101 | 1、DISCERN 2、PEMAT | 1. The videos scored highly in terms of comprehensibility (average of 78.8%), but scored lower in terms of actionability (average of 43.1%) and information reliability and quality (DISCERN average of 2.44/5).  2. The average score for actionability (PEMAT actionability) was 43.1%. |
| 22 | Abramson M  et al.[22]  2022 | Prostate Cancer Screening | 2021/8/12 | 80 | 1、DISCERN 2、PEMAT | 1. Insufficient video quality (DISCERN median score of only 36.5–43 out of 80)  2. Commercial bias present in 15–23% of cases |
| 23 | Gulve ND  et al.[23]  2022 | Oral Self-examination to Detect Oral Cancer  Meningiomas: | 2021/9/10 | 24 | 1、GQS 2、mDISCERN | 1. The mean GQS, DISCERN, and comprehensiveness scores for the evaluated videos were 3.71 ± 1.30, 3.71 ± 0.75, and 9 ± 1.89, respectively, indicating moderate quality. |
| 24 | Kaplan K  et al.[24]  2023 | Hepatocellular carcinoma | 2022/3/15 | 143 | 1、GQS 2、modified DISCERN | 1. Videos with 89.6% useful content received higher GQS scores (median 4 vs. 3) and DISCERN scores (median 4 vs. 1) (p < 0.001),  2. However, useful videos actually had lower view counts (median 1,551 vs. 3,720, p = 0.049). |
| 25 | Di Bello F  et al.[25]  2022 | Immunotherapy for Urological Tumors | 2022/3/25 | 156 | 1、PEMAT 2、DISCERN 3、Misinformation scale | 1. YouTube cannot be considered a reliable source of information on IMT for urological malignancies.  2. The DISCERN score was 44 (IQR: 39–53.2), and the overall Understandability score was 40% (Inter-Quartile Range [IQR]: 20–61.5). There was a statistically significant positive correlation between length and understandability (r = 0.31, p = 0.001). |
| 26 | Reinhardt L  et al.[26]  2023 | Skin cancer | 2022/5/1 | 38 | 1、DISCERN 2、JAMA 3、PEMAT 4、GQS | 1. The videos scored at a moderate to good level in terms of comprehensibility (average 64.27%), but their quality (DISCERN average 3.1/5), practicality (average 58.22%), and reliability (JAMA average 37.17%) were all relatively low. |
| 27 | Kang E  et al.[27]  2024 | Thyroid cancer | 2022/5/1 | 326 | 1、JAMAS 2、GQS 3、mDISCERN | 1. All videos received perfect scores on the PEMAT comprehensibility metric.  2. Although videos from public interest organizations and companies had the highest average view count (26,686.8 ± 156,763.4 views), their quality scores were lower. |
| 28 | Chen Z  et al.[28]  2023 | Anal Cancer and Its Precursor Lesions | 2022/5/29 | 105 | 1、DISCERN 2、JAMA 3、PEMAT 4、GQS | 1. Video DISCERN total score: 41.40 (range 16.00–77.00), JAMA: 2.94 (range 1.00–4.00), PEMAT understandability score: 87.77% (range 50.00%–100%), 48.73% (range 0–100%) and GQS 3.09 (range 1.00–5.00); video source was significantly correlated with scale scores (P < 0.001) |
| 29 | Baqain L  et al.[29]  2023 | Prostate cancer | 2022/6/26-2022/10/27 | 100 | 1、GQS 2、mDISCERN | 1. 84% of the videos contain useful content, 6% are misleading, 6% are irrelevant, and 4% are based on personal experiences; the quality is acceptable. |
| 30 | Jeong SY  et al.[30]  2026 | Ovarian cancer | 2022/10/4 | 86 | 1、GQS 2、mDISCERN | 1. The average educational quality score for the videos was 3.5 ± 1.1 (moderate level), but the average information reliability score was 2.2 ± 1.1 (low level); furthermore, the reliability of videos from medical sources was significantly higher than that of videos from non-medical sources (p = 0.001). |
| 31 | Lin ME  et al.[31]  2024 | Laryngectomy | 2022/11/1 | 78 | 1、DISCERN | 1. The overall quality of the videos was poor (mean DISCERN score of 1.97, SD = 1.12, rated as “poor”)  2. The quality of surgical videos was significantly lower than that of non-surgical videos (β = -1.63, 95% CI [-2.03, -1.24], p < 0.001).  3. A higher number of channel subscribers was significantly associated with improved video quality (β = 0.02, 95% CI [0.01, 0.03], p = 0.005). |
| 32 | Segado  et al.[32]  2023 | Cancer and Diet | 2022/12/1 | 72 | 1、mDISCERN 2、GQS | 1. The overall reliability and quality of the videos are low (average DISCERN score of 2.25 ± 0.88, average GQS score of 2.21 ± 1.11).  2. Only 20.8% were uploaded by health-related users, and 12.5% of the videos falsely claimed that cancer could be cured solely by eating “real food.” |
| 33 | Fernandez  et al.[33]  2024 | Lymphedema | 2023/1/1 | 35 | 1、DISCERN | 1. Videos posted by healthcare professionals scored significantly higher on reliability (p < 0.017) and total DISCERN scores (p < 0.03) than those from other sources;  2. Video length was positively correlated with total DISCERN scores (r = 0.476, p = 0.004). |
| 34 | Rodriguez  et al.[34]  2024 | Incontinence Information after Cancer Surgery | 2023/3/22 | 108 | 1、DISCERN 2、GQS 3、PEMAT 4、MQ-VET | 1. The overall video quality was rated as “high” (DISCERN average: 3.37/5; GQS average: 3.23/5).  2. There was a strong positive correlation among the five quality assessment scales (correlation coefficients ranging from 0.697 to 0.926, p < 0.01). |
| 35 | Kocamaz D  et al.[35] | Aerobic Exercise in Rehabilitation of Lung Cancer | 2023/5/27-28 | 150 | 1、DISCERN | 1. Only 12 videos met the relevant criteria for aerobic exercise rehabilitation for lung cancer. The median scores for video reliability and quality were 3 (range 2–5) and 3 (range 2–4), respectively. While high-quality videos were mostly uploaded by doctors, they generally lacked authoritative references and specific exercise parameters. |
| 36 | Sahin E  et al.[36]  2025 | Chemotherapy | 2023/8/25 | 108 | 1、mDISCERN 2、JAMA 3、GQS 4、VIQI 5、HONcode | 1. The quality and reliability of the video content were rated as slightly below average, with mean scores of 2.73 ± 1.18 for mDISCERN, 1.97 ± 1.05 for JAMA, 2.94 ± 1.08 for GQS, 14.03 ± 3.73 for VIQI, and 4.68 ± 2.46 for HONcode. |
| 37 | Liu Z  et al.[37]  2024 | laryngeal carcinoma | 2024/1/1 | 99 | 1、PEMAT 2、VIQI 3、GQS 4、mDISCERN | 1. Video quality was the best (median VIQI-sum of 15 [13, 16], median GQS of 3 [3, 4], and median mDISCERN of 3 [2, 4], all significantly higher than those of Bilibili and TikTok, p < 0.05), but overall quality still needs improvement. |
| 38 | Altintas T  et al.[38]  2025 | Prophylactic Mastectomy | 2024/2/1 | 50 | 1、DISCERN 2、GQS | 1. The average DISCERN score for the videos was 3.35 ± 1 (moderate reliability), and the average GQS score was 3.39 ± 0.9 (moderate quality). Specifically, videos uploaded by doctors were of good quality, those from health channels were of moderate quality, while videos uploaded by patients were of poor quality and misleading. |
| 39 | Bu Bshait  et al.[39]  2024 | Breast Self-Examination | 2024/3/11 | 50 | 1、JAMA 2、GQS | 1. The videos are relatively comprehensive in terms of specific instructional content (average score of 7.02 out of 9), but their reliability and overall educational quality are poor (average JAMA score of 1.00 out of 4, average GQS score of 3.24 out of 5). |
| 40 | Özeller E  et al.[40]  2025 | HPV Vaccination | 2024/5/1 | 81 | 1、PEMAT 2、JAMA 3、VPI 4、VIQI 5、GQS | 1. The videos are of moderate quality (median GQS score of 3/5), with low transparency and reliability (median JAMA score of 2/4), and lack the involvement of family physicians. |
| 41 | Sözlü S  et al.[41]  2026 | Pediatric Cancer | 2024/5/14 | 25 | 1、mDISCERN 2、GQS | 1. Only 60% of the videos were classified as useful, while 40% were deemed misleading; furthermore, the viewership of reliable videos was significantly lower than that of unreliable videos (p < 0.05). |
| 42 | Zhang Q  et al.[42]  2025 | Oral cancer | 2024/8/7 | 150 | 1、PEMAT 2、VIQI 3、GQS 4、mDISCERN | 1. The videos significantly outperformed Bilibili in terms of understandability (PEMAT-Understandability: 75.1), information quality (VIQI-sum: 14.1), and overall quality (GQS: 3.0). However, there was no significant difference between the two platforms in terms of actionability (PEMAT-Actionability) and reliability (mDISCERN). |
| 43 | Mezzapesa F  et al.[43]  2025 | Current Use of HIPEC in the Treatment of Ovarian Cancer | 2024/8/31 | 127 | 1、PEMAT 2、DISCERN 3、GQS | 1. The video quality for the general public was significantly lower than that for healthcare professionals (median DISCERN total score of 40 vs. 56, p < 0.001), and the overall usability score was only 33%. |
| 44 | Dusunceli I  et al.[44]  2026 | Colonoscopy | 2024/10/-23至30 | 156 | 1、mDISCERN 2、JAMA 3、GQS | 1. The overall scientific quality of the videos ranged from low to moderate (mDISCERN scores: 24.4% poor, 59% moderate, 16.7% good);  2. Only 12.8% met the JAMA criteria; the average C-DQS score was 7.1 out of 40. |
| 45 | Marschner F  et al.[45]  2026 | dental care in head and neck cancer | 2025/2/15-18 | 134 | 1、LIDA 2、FRES 3、DISCERN | 1. The comprehensiveness of video content is relatively low (median 7.0, with no significant difference from the website, p=0.924), and viewer engagement is low (median 0.9%); overall, the quality is unreliable. |
| 46 | Qi S  et al.[46]  2025 | Cervical cancer | 2025/2/21 | 84 | 1、GQS 2、VIQI 3、mDISCERN 4、PEMAT | 1. The median GQS score was 4 (Good), the median VIQI was 14, the median mDISCERN score was 3, and the median PEMAT scores were 84.5% for comprehensibility and 33% for actionable content. Most videos were uploaded by healthcare institutions (47.76%), and the overall content quality and reliability were significantly higher than those on TikTok. |
| 47 | Zhao CY  et al.[47]  2026 | Uterine fibroids | 2025/3/18-21 | 100 | 1、JAMA 2、mDISCERN 3、GQS | 1. The overall quality of videos related to uterine fibroids was moderate to low (median GQS score of 2, median DISCERN score of 3), and video length was positively correlated with quality (Spearman’s ρ ≈ 0.33, p < 0.01). |
| 48 | Sandıkcı F  et al.[48]  2026 | Prostate biopsy | 2025/5/18 | 50 | 1、GQS 2、mDISCERN | 1. The average GQS score for the videos was 2.96 ± 0.77 (moderate quality), and the average mDISCERN score was 2.1 ± 0.9 (low to moderate reliability). Although 60% were produced by healthcare professionals, none cited peer-reviewed literature, and only 18% were free of commercial bias. |
| 49 | Gülmez H  et al.[49]  2026 | Human Papillomavirus and Vaccines | 2025/7/1-31 | 270 | 1、VPI 2、DISCERN 3、JAMA | 1. The videos had an average DISCERN score of 47.6 ± 15.1 (moderate quality) and an average JAMA score of 1.9 ± 0.9 (low reliability). |
| 50 | Liu Y  et al.[50]  2026 | Cervical cancer | 2025/7/1 | 70 | 1、GQS 2、mDISCERN 3、PEMAT 4、JAMA | 1. The videos had the highest quality (mean GQS of 3.47 ± 1.06) and were significantly more understandable than those on TikTok (76.94 ± 10.43 vs. 70.14 ± 11.07, p < 0.001); however, coverage of key preventive content (such as HPV vaccination) remained insufficient. |
| 51 | Xu T  et al.[51]  2025 | Vulvar cancer | 2025/9/5 | 65 | 1、GQS 2、DISCERN 3、DISCERN | 1. The average DISCERN score for the videos was 40.78 (out of 80), with only 13.85% of the videos rated as “Excellent,” indicating that the quality was subpar. |
| 52 | Soroudi D  et al.[52]  2024 | oncologic breast reconstruction | Published on 2024/7/23 | 172 | 1、mDISCERN | 1. DISCERN scores for academic sources (healthcare institutions and physicians) were significantly higher than those for non-academic sources (news media and patients) (2.98 ± 0.95 vs. 2.28 ± 0.77, p < 0.001); overall quality was moderate. |
| 53 | Yurdaisik I  et al.[53]  2020 | Breast cancer | Published on 2020/5/25 | 50 | 1、DISCERN 2、JAMA | 1. The mean DISCERN score was 26.70 ± 10.99; the mean JAMA score was 2.23 ± 0.97. For 66% of the videos, the DISCERN score was <28 (very poor); for 20%, it was poor (28–38); for 12%, it was average (39–50); and for 2%, it was excellent (63–75). Overall, video quality was poor (66% very poor).  2. Videos uploaded by physicians had significantly higher DISCERN scores than those uploaded by non-physicians (33.86 vs. 25.51, p=0.001). |
| 54 | Loeb S  et al.[54]  2019 | Prostate cancer | Published on  2019/4 | 150 | 1、DISCERN | 1. 77% of the videos (reaching over 6 million viewers) contained potentially misleading or biased content either in the video itself or in the comments section.  2. There was a significant negative correlation between the scientific quality score of the videos and viewer engagement (monthly average views and the like-to-view ratio) (p=0.004 and p=0.015). |

Abbreviations: C-DQS, Colonoscopy Data Quality Score; CI, confidence interval; DISCERN, an instrument for assessing the quality of consumer health information on treatment choices; FRES, Flesch Reading Ease Score; GQS, Global Quality Score; HIPEC, hyperthermic intraperitoneal chemotherapy; HONcode, Health On the Net Code of Conduct; HPV, human papillomavirus; IQR, interquartile range; JAMA, Journal of the American Medical Association benchmark criteria; LAM, lymphangioleiomyomatosis; LIDA, an instrument for evaluating the accessibility, usability, and reliability of online health information; mDISCERN, modified DISCERN; MICI, Medical Information and Content Index; MQ-VET, Medical Quality Video Evaluation Tool; PEMAT, Patient Education Materials Assessment Tool; SD, standard deviation; VIQI, Video Information and Quality Index; VPI, Video Power Index.

**References**

[1] Aydin MA, Akyol H. Quality of Information Available on YouTube Videos Pertaining to Thyroid Cancer. J Cancer Educ. 2020 Jun;35(3):599-605. doi: 10.1007/s13187-019-01502-9.
[2] Esen E, Aslan M, Sonbahar BÇ, Kerimoğlu RS. YouTube English videos as a source of information on breast self-examination. Breast Cancer Res Treat. 2019 Feb;173(3):629-635. doi: 10.1007/s10549-018-5044-z.
[3] Korkmaz U, Soyluoglu S, Arda E. Radionuclide Therapy Videos on YouTube as An Educational Material: Has the COVID-19 Pandemic Changed the Quality, Usefulness, and Interaction Features. Nucl Med Mol Imaging. 2023 Apr 5;57(4):1-7. doi: 10.1007/s13139-023-00799-1.
[4] Rodriguez Rodriguez AM, Blanco-Díaz M, Lopez Diaz P, de la Fuente Costa M, Dueñas L, Escobio Prieto I, Calatayud J, Casaña J. Quality Analysis of YouTube Videos Presenting Shoulder Exercises after Breast Cancer Surgery. Breast Care (Basel). 2022 Apr;17(2):188-198. doi: 10.1159/000518265.
[5] Zhang S, Fukunaga T, Oka S, Orita H, Kaji S, Yube Y, Yamauchi S, Kohira Y, Egawa H. Concerns of quality, utility, and reliability of laparoscopic gastrectomy for gastric cancer in public video sharing platform. Ann Transl Med. 2020 Mar;8(5):196. doi: 10.21037/atm.2020.01.78.
[6] Shungu N, Haley SP, Berini CR, Foster D, Diaz VA. Quality of YouTube Videos on Prostate Cancer Screening for Black Men. J Am Board Fam Med. 2021 Jul-Aug;34(4):724-731. doi: 10.3122/jabfm.2021.04.200632.
[7] Wilkens FM, Ganter C, Kriegsmann K, Wilkens H, Kahn N, Goobie GC, Ryerson CJ, Kreuter M. YouTube-videos for patient education in lymphangioleiomyomatosis? Respir Res. 2022 Apr 27;23(1):103. doi: 10.1186/s12931-022-02022-9.
[8] Ayoub G, Chalhoub E, Sleilaty G, Kourie HR. YouTube as a source of information on breast cancer in the Arab world. Support Care Cancer. 2021 Dec;29(12):8009-8017. doi: 10.1007/s00520-021-06403-6.
[9] Steeb T, Reinhardt L, Harlaß M, Heppt MV, Meier F, Berking C. Assessment of the Quality, Understandability, and Reliability of YouTube Videos as a Source of Information on Basal Cell Carcinoma: Web-Based Analysis. JMIR Cancer. 2022 Mar 11;8(1):e29581. doi: 10.2196/29581.
[10] Richardson MA, Park W, Bernstein DN, Mesfin A. Analysis of the Quality, Reliability, and Educational Content of YouTube Videos Concerning Spine Tumors. Int J Spine Surg. 2022 Apr;16(2):278-282. doi: 10.14444/8215.
[11] Cakmak G, Mantoglu B. Reliability and Quality of YouTube Contents Pertaining to Pancreatic Cancer. Cureus. 2021 Mar 24;13(3):e14085. doi: 10.7759/cureus.14085.
[12] Kahlam A, Pai S, Kahlam J, Ahlawat S. Quality of YouTube Videos Related to Colorectal Cancer Screening. Cureus. 2023 Jan 12;15(1):e33684. doi: 10.7759/cureus.33684.
[13] Yalkin O, Uzunoglu MY, İflazoglu N, Esen E. Are YouTube Videos a Reliable Source for Obtaining Information About Colorectal Cancer Screening? Cureus. 2022 Jan 24;14(1):e21550. doi: 10.7759/cureus.21550.
[14] Ekmez F, Ekmez M. Evaluation of the Quality and Reliability of YouTube Videos With Turkish Content as an Information Source for Gynecological Cancers During the COVID-19 Pandemic. Cureus. 2023 Sep 2;15(9):e44581. doi: 10.7759/cureus.44581.
[15] Gad B, Shanmugasundaram S, Kumar A, Shukla P. Quality and Reliability of YouTube Videos on Uterine Fibroid Embolization. J Am Coll Radiol. 2022 Jul;19(7):905-912. doi: 10.1016/j.jacr.2022.03.014.
[16] Duran MB, Kizilkan Y. Quality analysis of testicular cancer videos on YouTube. Andrologia. 2021 Sep;53(8):e14118. doi: 10.1111/and.14118.
[17] Bahar-Ozdemir Y, Ozsoy-Unubol T, Akyuz G. Is YouTube a high-quality source of information on cancer rehabilitation? J Cancer Surviv. 2022 Oct;16(5):1016-1022. doi: 10.1007/s11764-021-01093-9
[18] Vu E, Pratsinis M, Plasswilm L, Schmid HP, Panje C, Betschart P. Radiotherapy or Surgery? Comparative, Qualitative Assessment of Online Patient Education Materials on Prostate Cancer. Curr Oncol. 2021 Sep 6;28(5):3420-3429. doi: 10.3390/curroncol28050296.
[19] García-Cano-Fernández AM, Szczesniewski-Dudzik JJ, García-Tello A, Diego-García V, Boronat-Catalá J, Llanes-González L. Quality of bladder cancer information on YouTube. Cent European J Urol. 2022;75(3):248-251. doi: 10.5173/ceju.2022.151.
[20] Krakowiak M, Fercho J, Piwowska K, Yuser R, Szmuda T, Słoniewski P. YouTube as a Source of Patient Information for Meningiomas: A Content Quality and Audience Engagement Analysis. Healthcare (Basel). 2022 Mar 10;10(3):506. doi: 10.3390/healthcare10030506.
[21] Morena N, Ahisar Y, Wang X, Nguyen D, Rentschler CA, Meguerditchian AN. Content Quality of YouTube Videos About Metastatic Breast Cancer in Young Women: Systematic Assessment. JMIR Cancer. 2023 Nov 14;9:e45101. doi: 10.2196/45101.
[22] Abramson M, Feiertag N, Javidi D, Babar M, Loeb S, Watts K. Accuracy of prostate cancer screening recommendations for high-risk populations on YouTube and TikTok. BJUI Compass. 2022 Nov 8;4(2):206-213. doi: 10.1002/bco2.200.
[23] Gulve ND, Tripathi PR, Dahivelkar SD, Gulve MN, Gulve RN, Kolhe SJ. Evaluation of YouTube Videos as a Source of Information About Oral Self-examination to Detect Oral Cancer and Precancerous Lesions. J Int Soc Prev Community Dent. 2022 Apr 8;12(2):226-234. doi: 10.4103/jispcd.JISPCD_277_21.
[24] Kaplan K, Solak Y. Evaluation of YouTube Videos on Hepatocellular Carcinoma. J Korean Med Sci. 2023 Feb 20;38(7):e50. doi: 10.3346/jkms.2023.38.e50.
[25] Di Bello F, Di Mauro E, Collà Ruvolo C, Creta M, La Rocca R, Celentano G, Capece M, Napolitano L, Fraia A, Pezone G, Morra S, Imbimbo C, Mirone V, Longo N, Califano G. Immunotherapy for Urological Tumors on YouTubeTM: An Information-Quality Analysis. Vaccines (Basel). 2022 Dec 30;11(1):92. doi: 10.3390/vaccines11010092.
[26] Reinhardt L, Steeb T, Mifka A, Berking C, Meier F; German Skin Cancer Council. Quality, Understandability and Reliability of YouTube Videos on Skin Cancer Screening. J Cancer Educ. 2023 Oct;38(5):1667-1674. doi: 10.1007/s13187-023-02320-w.
[27] Kang E, Ju H, Kim S, Choi J. Contents analysis of thyroid cancer-related information uploaded to YouTube by physicians in Korea: endorsing thyroid cancer screening, potentially leading to overdiagnosis. BMC Public Health. 2024 Apr 2;24(1):942. doi: 10.1186/s12889-024-18403-2.
[28] Chen Z, Pan S, Zhou M, Wang X. Evaluation of the quality and reliability of anal cancer and its precancerous lesions-related content on YouTube: a cross-sectional study. BMJ Open. 2023 Oct 18;13(10):e074787. doi: 10.1136/bmjopen-2023-074787.
[29] Baqain L, Mukherji D, Al-Shamsi HO, Abu-Gheida I, Ibraheem AA, Rabii KA, Farkouh A, Shahait M. Quality and reliability of YouTube videos in Arabic as a source of patient information on prostate cancer. Ecancermedicalscience. 2023 Jul 13;17:1573. doi: 10.3332/ecancer.2023.1573.
[30] Jeong SY, Kim J, Hwang J, Park ST, Park SH. The Role of YouTube as an Information Platform for Ovarian Cancer: the Educational Quality and Reliability. Yonsei Med J. 2026 Jun;67(6):502-507. doi: 10.3349/ymj.2025.0374.
[31] Lin ME, Ayo-Ajibola O, Castellanos CX, West J, Luu N, Kim I, Kokot NC. Assessing Laryngectomy Patient Education on YouTube: Investigating Quality and Reliability. OTO Open. 2024 Jan 31;8(1):e113. doi: 10.1002/oto2.113.
[32] Segado-Fernández S, Herrera-Peco I, Jiménez-Gómez B, Ruiz Núñez C, Jiménez-Hidalgo PJ, Benítez de Gracia E, González-Rodríguez LG, Torres-Ramírez C, Lozano-Estevan MDC. Realfood and Cancer: Analysis of the Reliability and Quality of YouTube Content. Int J Environ Res Public Health. 2023 Mar 13;20(6):5046. doi: 10.3390/ijerph20065046.
[33] Fernandez-Alvarez MDM, Cachero-Rodríguez J, Fu MR, Sánchez-Fernández P, Ureña-Lorenzo A, Martin-Payo R. YouTube videos on lymphedema as an information source for Spanish speaking breast cancer survivors. Support Care Cancer. 2024 Jul 24;32(8):540. doi: 10.1007/s00520-024-08746-2.
[34] Rodriguez-Rodriguez AM, De la Fuente-Costa M, Escalera-de la Riva M, Domínguez-Navarro F, Perez-Dominguez B, Paseiro-Ares G, Casaña-Granell J, Blanco-Diaz M. Assessing the Quality of YouTube's Incontinence Information after Cancer Surgery: An Innovative Graphical Analysis. Healthcare (Basel). 2024 Jan 18;12(2):243. doi: 10.3390/healthcare12020243 .
[35] Kocamaz D, Demircioğlu Karagöz A, Atasavun Uysal S. YouTube Videos as an Information Source About Aerobic Exercise in Rehabilitation of Lung Cancer. Integr Cancer Ther. 2025 Jan-Dec;24:15347354251331461. doi: 10.1177/15347354251331461.
[36] Sahin E, Seyyar M. Assessing the scientific quality and reliability of YouTube videos about chemotherapy. Medicine (Baltimore). 2023 Nov 10;102(45):e35916. doi: 10.1097/MD.0000000000035916.
[37] Liu Z, Chen Y, Lin Y, Ai M, Lian D, Zhang Y, Shen Y, Yu F. YouTube/ Bilibili/ TikTok videos as sources of medical information on laryngeal carcinoma: cross-sectional content analysis study. BMC Public Health. 2024 Jun 14;24(1):1594. doi: 10.1186/s12889-024-19077-6.
[38] Altintas T, Bilgi Kirmaci M. Evaluating YouTube Videos With Prophylactic Mastectomy Content in Terms of Their Quality and Reliability. Breast J. 2025 Jan 9;2025:9487931. doi: 10.1155/tbj/9487931.
[39] Bu Bshait MS, Almaqhawi A. Quality Assessment of YouTube Videos As Information Source for Breast Self-Examination. Cureus. 2024 Sep 26;16(9):e70227. doi: 10.7759/cureus.70227.
[40] Özeller E, Toçoğlu M, Aktı-Çakır E, Hüsna SÇ. Analyzing Turkish-Language HPV Vaccination Videos on YouTube: Assessing Content Quality and Educational Value. Infect Dis Clin Microbiol. 2025 Sep 25;7(3):310-319. doi: 10.36519/idcm.2025.699.
[41] Sözlü S, Macit-Çelebi MS, Kocaadam-Bozkurt B. YouTube videos on nutrition and pediatric cancer: scientific reliability and quality. Front Public Health. 2026 Feb 27;14:1767637. doi: 10.3389/fpubh.2026.1767637.
[42] Zhang Q, Li Z, Zhang H, Han L, Zhao S, Jia S. YouTube and Bilibili as sources of information on oral cancer: cross-sectional content analysis study. Sci Rep. 2025 Jul 1;15(1):21671. doi: 10.1038/s41598-025-02898-9.
[43] Mezzapesa F, Bilancia EP, Afonina M, Di Costanzo S, Masina E, De Iaco P, Perrone AM. Is YouTube™ a Reliable Source of Information for the Current Use of HIPEC in the Treatment of Ovarian Cancer? Cancers (Basel). 2025 Oct 2;17(19):3222. doi: 10.3390/cancers17193222.
[44] Dusunceli I, Sargin ZG, Sargin F. Scientific quality of colonoscopy-related YouTube videos in the Turkish language. Medicine (Baltimore). 2026 Apr 3;105(14):e48231. doi: 10.1097/MD.0000000000048231.
[45] Marschner F, Runkel L, Range M, Lechte C, Wiegand A. How Reliable is eHealth information on dental care in head and neck cancer? A quality evaluation. BMC Oral Health. 2026 Apr 29;26(1):814. doi: 10.1186/s12903-026-08488-z..
[46] Qi S, Chen Q, Liang J, Yang X, Li X. Evaluating the quality and reliability of cervical cancer related videos on YouTube, Bilibili, and Tiktok: a cross-sectional analysis. BMC Public Health. 2025 Oct 31;25(1):3682. doi: 10.1186/s12889-025-24840-4.
[47] Zhao CY, Wang YP, Jin L, Zhao XL, Yang XK. TikTok, YouTube, and Bilibili as sources of information on uterine fibroids: A content and quality analysis. Medicine (Baltimore). 2026 Mar 13;105(11):e48063. doi: 10.1097/MD.0000000000048063.
[48] Sandıkcı F, Altan M. Quality, reliability, and content analysis of YouTube videos on transperineal prostate biopsy. Digit Health. 2026 Mar 23;12:20552076261432019. doi: 10.1177/20552076261432019.
[49] Gülmez H, Ciftci M. Quality, reliability and popularity assessment of Turkish YouTube videos on human papillomavirus and its vaccine: content analysis. PeerJ. 2026 Feb 18;14:e20828. doi: 10.7717/peerj.20828.
[50] Liu Y, Wu R, Zhang L. A cross-sectional study on the quality of cervical cancer health information across multiple short video platforms: Analysis of content, quality, and dissemination characteristics. Digit Health. 2026 Jan 21;12:20552076261415927. doi: 10.1177/20552076261415927.
[51] Xu T, Zhao H. Analysis of the quality of vulvar cancer-related videos on YouTube. Transl Cancer Res. 2025 Jan 31;14(1):102-111. doi: 10.21037/tcr-24-1411.
[52] Soroudi D, Falade I, Mehta N, Gursky A, Yin R, Song S, Kim E. Evaluating the quality and reliability of oncologic breast reconstruction videos on youtube. JPRAS Open. 2024 Oct 11;42:311-314. doi: 10.1016/j.jpra.2024.10.006.
[53] Yurdaisik I. Analysis of the Most Viewed First 50 Videos on YouTube about Breast Cancer. Biomed Res Int. 2020 May 25;2020:2750148. doi: 10.1155/2020/2750148.
[54] Loeb S, Sengupta S, Butaney M, Macaluso JN Jr, Czarniecki SW, Robbins R, Braithwaite RS, Gao L, Byrne N, Walter D, Langford A. Dissemination of Misinformative and Biased Information about Prostate Cancer on YouTube. Eur Urol. 2019 Apr;75(4):564-567. doi: 10.1016/j.eururo.2018.10.056.
