# Supplementary material for: Quality and reliability of cancer- and tumor-related videos on YouTube: a scoping review
Source: Front Public Health. 2026 Oct 14;14:1940232. doi: 10.3389/fpubh.2026.1940232 (PMC13713483; doi:10.3389/fpubh.2026.1940232)
Supplement: Supplementary file 2 [file Supplementary_Table_4.docx]

**English search terms:**

subject heading：Neoplasms

free-text term ：Cancer，Tumor，Tumour，carcinoma，Malignancy，oncology

subject headings+free-text term ：Neoplasms OR Cancer OR Tumor OR Tumor OR carcinomaOR Malignancy OR oncology

free-text term： reliability OR credibility

free-text term：Quality Assessment OR Quality Evaluation OR Information Quality

free-text term：DISCERN OR modified DISCERN OR GQS OR Global Quality ScaleOR PEMAT OR VIQI OR JAMA OR JAMA benchmark

**Search the database**

**PubMed**

Search: **("YouTube"[Title/Abstract])AND( "Neoplasms"[Mesh] OR "cancer"[Title/Abstract] OR "tumor"[Title/Abstract] OR "tumour"[Title/Abstract] OR "carcinoma"[Title/Abstract] OR "malignancy"[Title/Abstract] OR "oncology"[Title/Abstract])AND("quality assessment"[Title/Abstract] OR "quality evaluation"[Title/Abstract] OR "information quality"[Title/Abstract] OR "reliability"[Title/Abstract] OR "credibility"[Title/Abstract] OR "DISCERN"[Title/Abstract] OR"modified DISCERN"[Title/Abstract] OR "GQS"[Title/Abstract] OR "Global Quality Scale"[Title/Abstract] OR "PEMAT"[Title/Abstract] OR "VIQI"[Title/Abstract] OR "JAMA"[Title/Abstract] OR "JAMA benchmark"[Title/Abstract )**

**Cochrane Library**

(Cancer):ti,ab,kw OR (Tumor):ti,ab,kw OR (Tumour):ti,ab,kw OR (carcinoma):ti,ab,kw OR (Malignancy):ti,ab,kw OR (oncology):ti,ab,kw

(reliability):ti,ab,kw OR (credibility):ti,ab,kw

(Quality Assessment):ti,ab,kw OR (Quality Evaluation):ti,ab,kw OR (Information Quality):ti,ab,kw

(DISCERN):ti,ab,kw OR (modified DISCERN):ti,ab,kw OR (GQS):ti,ab,kw OR (Global Quality Scale):ti,ab,kw OR (PEMAT):ti,ab,kw OR (VIQI):ti,ab,kw OR (JAMA):ti,ab,kw OR (JAMA benchmark):ti,ab,kw

**Scopus**

TITLE-ABS-KEY("YouTube") AND TITLE-ABS-KEY("Neoplasms" OR "cancer" OR "tumor" OR "tumour" OR "carcinoma" OR "malignancy" OR "oncology") AND TITLE-ABS-KEY("quality assessment" OR "quality evaluation" OR "information quality" OR "reliability" OR "credibility") AND TITLE-ABS-KEY("DISCERN" OR "modified DISCERN" OR "GQS" OR "Global Quality Scale" OR "PEMAT" OR "VIQI" OR "JAMA" OR "JAMA benchmark")

**Embase**

‘Cancer’:ti,ab OR ‘Tumor’:ti,ab OR ‘Tumour’:ti,ab OR ‘carcinoma’:ti,ab OR ‘Malignancy’:ti,ab OR ‘oncology’:ti,ab

‘reliability’:ti,ab OR ‘credibility’:ti,ab

‘Quality Assessment’:ti,ab OR ‘Quality Evaluation’:ti,ab OR ‘Information Quality’:ti,ab

‘DISCERN’:ti,ab OR ‘modified DISCERN’:ti,ab OR ‘GQS’:ti,ab OR ‘Global Quality Scale’:ti,ab OR ‘PEMAT’:ti,ab OR ‘VIQI’:ti,ab OR ‘JAMA’:ti,ab OR ‘JAMA benchmark’:ti,ab

**CINAHL**

XB (Neoplasms OR Cancer OR Tumor OR Tumour OR carcinomaOR Malignancy OR oncology) AND XB (reliability OR credibility) AND XB (Quality Assessment OR Quality Evaluation OR Information Quality) AND XB (DISCERN OR modified DISCERN OR GQS OR Global Quality ScaleOR PEMAT OR VIQI OR JAMA OR JAMA benchmark)

**Web of science**

TS=（Neoplasms OR Cancer OR Tumor OR Tumour OR carcinomaOR Malignancy OR oncology）

TS=（reliability OR credibility）

TS=（Quality Assessment OR Quality Evaluation OR Information Quality）

TS=（DISCERN OR modified DISCERN OR GQS OR Global Quality ScaleORPEMAT OR VIQI OR JAMA OR JAMA benchmark）

Chinese Search Term

（1）YouTube

（2）肿瘤 + 癌症 + 癌 + 瘤 + 恶性肿瘤

（2）可靠性 + 可信度

（3）质量评估 + 质量评价 + 信息质量

（1）肿瘤 or 癌症 or 癌 or 瘤 or 恶性肿瘤

（2）可靠性 or 可信度

（3）质量评估 or 质量评价 or 信息质量

**CNKI**

(篇关摘='YouTube')

AND

(篇关摘='肿瘤' OR '癌症' OR '癌' OR '恶性肿瘤' OR '瘤')

AND

(篇关摘='质量评价' OR '质量评估' OR '信息质量' OR '可靠性' OR '可信度' OR 'DISCERN' OR '改良DISCERN' OR 'GQS' OR '全球质量评分' OR 'PEMAT' OR 'VIQI' OR 'JAMA' OR 'JAMA基准')

VIP

(T=YouTube OR K=YouTube OR R=YouTube) AND (T=癌症 OR K=癌症 OR T=肿瘤 OR K=肿瘤 OR R=癌症 OR R=肿瘤) AND (T=质量 OR K=质量 OR T=可靠性 OR K=可靠性 OR T=评价 OR K=评价 OR T=评估 OR K=评估 OR R=质量 OR R=可靠性 OR R=评价 OR R=评估)
